# Systematic analysis of Mendelian disease-associated gene variants reveals new classes of cancer-predisposing genes

**DOI:** 10.1186/s13073-023-01252-w

**Published:** 2023-12-25

**Authors:** Seulki Song, Youngil Koh, Seokhyeon Kim, Sang Mi Lee, Hyun Uk Kim, Jung Min Ko, Se-Hoon Lee, Sung-Soo Yoon, Solip Park

**Affiliations:** 1 Cancer Research Institute, Seoul National University College of Medicine, Seoul, 03080 Republic of Korea; 2 Structural Biology Program, Centro Nacional de Investigaciones Oncológicas (CNIO), Calle de Melchor Fernández Almagro, 3, Madrid, 28029 Spain; 3 Biomedical Research Institute and Departments of Internal Medicine, Seoul National University Hospital, Seoul, 03080 Republic of Korea; 4 Department of Chemical and Biomolecular Engineering, Korea Advanced Institute of Science and Technology (KAIST), Daejeon, 34141 Republic of Korea; 5 Department of Pediatrics, Seoul National University Children’s Hospital, Seoul National University College of Medicine, Seoul, 03080 Republic of Korea; 6 Division of Hematology-Oncology, Department of Medicine, Samsung Medical Center, Sungkyunkwan University School of Medicine, Seoul, 06351 Republic of Korea

**Keywords:** Mendelian disease-associated gene, Cancer predisposition gene, Pathogenic variant, Cancer genomics

## Abstract

**Background:**

Despite the acceleration of somatic driver gene discovery facilitated by recent large-scale tumor sequencing data, the contribution of inherited variants remains largely unexplored, primarily focusing on previously known cancer predisposition genes (CPGs) due to the low statistical power associated with detecting rare pathogenic variant-phenotype associations.

**Methods:**

Here, we introduce a generalized log-regression model to measure the excess of pathogenic variants within genes in cancer patients compared to control samples. It aims to measure gene-level cancer risk enrichment by collapsing rare pathogenic variants after controlling the population differences across samples.

**Results:**

In this study, we investigate whether pathogenic variants in Mendelian disease-associated genes (OMIM genes) are enriched in cancer patients compared to controls. Utilizing data from PCAWG and the 1,000 Genomes Project, we identify 103 OMIM genes demonstrating significant enrichment of pathogenic variants in cancer samples (FDR 20%). Through an integrative approach considering three distinct properties, we classify these CPG-like OMIM genes into four clusters, indicating potential diverse mechanisms underlying tumor progression. Further, we explore the function of *PAH* (a key metabolic enzyme associated with Phenylketonuria), the gene exhibiting the highest prevalence of pathogenic variants in a pan-cancer (1.8%) compared to controls (0.6%).

**Conclusions:**

Our findings suggest a possible cancer progression mechanism through metabolic profile alterations. Overall, our data indicates that pathogenic OMIM gene variants contribute to cancer progression and introduces new CPG classifications potentially underpinning diverse tumorigenesis mechanisms.

**Supplementary Information:**

The online version contains supplementary material available at 10.1186/s13073-023-01252-w.

## Background

Inherited genetic variants can substantially increase an individual's cancer risk. This concept of a genetic predisposition to cancer was initially proposed by Broca, who noted cases of breast cancer in 15 members of his wife’s family [1]. Building on this, Alfred Knudson proposed the ‘two-hit’ hypothesis in 1971, suggesting that tumorigenesis occurs when both alleles of a given gene are inactivated. In this model, an inherited variant, or the ‘first hit’, inactivates one allele. A subsequent ‘second hit’, which inactivates the remaining allele in somatic cells, then promotes tumorigenesis [2]. This ‘two-hit’ model has been exemplified in the case of the Retinoblastoma predisposition gene *RB1*, the first identified cancer predisposing gene (CPG) [3].

Since the initial discovery of the RB1 gene, over 100 CPGs have been identified, primarily through the detection of high-penetrance variants using linkage or candidate gene analysis in small-scale family studies [4]. Typically, CPGs are monogenic, ubiquitously expressed, and involved in essential cellular processes such as cell cycle regulation or DNA repair pathways [5]. More recently, the advent of large-scale next-generation whole-exome or genome sequencing analyses has led to the discovery of new CPG candidates [4]. Moreover, large-scale cancer genome sequencing has begun to reveal CPGs that operate through mechanisms distinct from the classical ‘two-hit’ model, including one-hit CPGs and tissue-specific CPGs [6, 7]. Nevertheless, the contribution of inherited variants is still underestimated due to the low statistical power associated with detecting rare pathogenic variant-phenotype associations (such as cancer risk), tissue-specific effects, and the low penetrance of certain inherited variants [4].

**Fig. 1:**
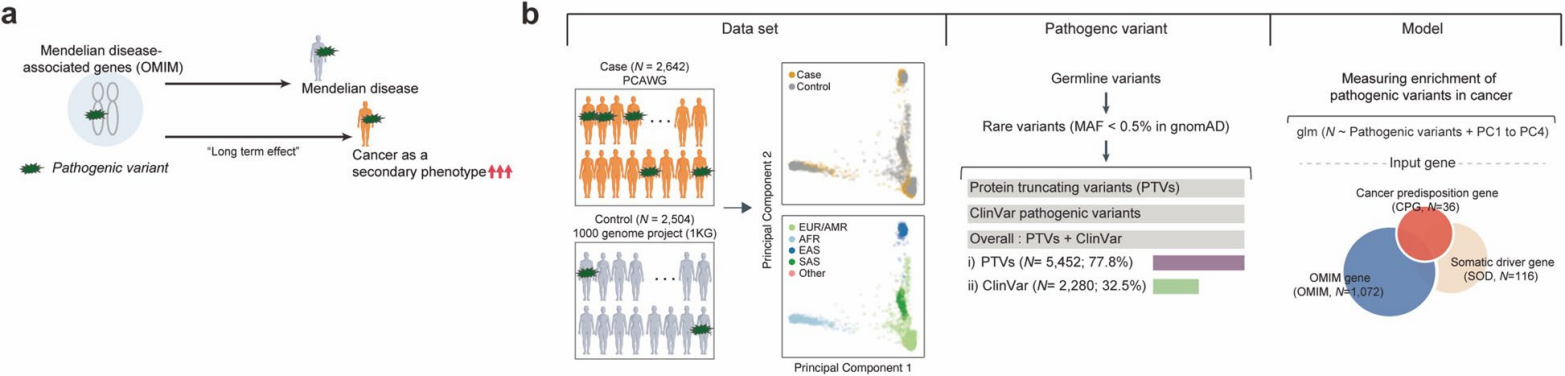
Systematic analysis of the enrichment of rare pathogenic variants in cases compared to control samples. **a** Proposed hypothesis that rare pathogenic variants in Mendelian disease-associated genes (OMIM genes) increase the risk of cancer. **b** Overview of the case–control analysis. Principal components analysis (PCA) using common variants was performed to stratify the population of cases (cancer patients) and control individuals. After defining pathogenic variants (PTVs or ClinVar pathogenic variants) from case–control samples, the linear regression model tests germline variant enrichments in cases compared to controls with the first four PC values. AFR: African, AMR: American, EAS: East Asian, EUR: European, and SAS: South Asian

To systematically identify potential novel CPGs, we focused on genes associated with Mendelian diseases as catalogued in the Online Mendelian Inheritance in Man (OMIM) database [8]. This database lists human genes linked with inheritable disorders, with information primarily based on genetic linkage studies. Similar to CPGs, OMIM genes are typically monogenic and exhibit high-penetrance phenotypes (Fig. 1a). It is noteworthy that patients carrying pathogenic variants in OMIM genes have been reported to exhibit secondary phenotypes in adulthood, including cancer [4, 9]. For instance, individuals with rare variants in the GBA gene, which is associated with Gaucher's disease in childhood, have an increased risk of developing Parkinson's disease and cancers, including multiple melanomas [10, 11]. These observations underscore the potential of pathogenic variants in OMIM genes to increase the susceptibility to other disorders, including cancer. Consequently, a systematic analysis of variants in OMIM genes could reveal new candidate CPGs.

In this study, we evaluated whether rare germline variants in OMIM genes contribute to cancer risk, serving as potentially novel CPGs. We hypothesized that: (i) pathogenic variants in OMIM genes are enriched in cancer patients compared to healthy controls, and (ii) these OMIM genes could provide insight into novel cancer-promoting mechanisms. To test these hypotheses, we undertook a systematic analysis of large-scale cancer genomic data from the Pan-cancer Analysis of Whole Genomes (PCAWG) project and the 1000 Genome project (1 KG), which represents healthy controls. Our analysis identified 103 OMIM genes displaying a significant excess of pathogenic variants in cancer patients compared to controls (FDR = 20%). Moreover, we classified the cancer-enriched OMIM genes into four distinct clusters based on the prevalence of bi-allelic inactivation, gene expression patterns, and loss-of-function tolerance. Among all the OMIM genes evaluated, pathogenic variants in *PAH* were the most prevalent in cancer patients compared to controls. As such, we conducted further analysis on *PAH* and found evidence suggesting that pathogenic variants in this gene elevate cancer risk by altering metabolic and immune response-associated pathways. Therefore, our investigation into variants in OMIM genes has identified novel candidate CPGs that potentially elucidate mechanisms underlying tumorigenesis.

## Methods

### Tumor sequencing data from PCAWG

The final consensus set of merged germline mutation calls in variant call format (VCF) generated by the Pan-cancer Analysis of Whole Genomes (PCAWG) [12] consortium was obtained from the International Cancer Genome Consortium Portal (https://dcc.icgc.org/releases/PCAWG) with authorization. The final dataset contains 2,642 high-quality samples after excluding 192 samples due to potential technical issues such as contamination or low-quality from the original pool 2,834 donors [12]. The germline variant calling file was divided into two germline VCF files: one containing 1,823 donors from the International Cancer Genome Consortium (ICGC), excluding US donors, and the other containing 819 donors from The Cancer Genome Atlas (TCGA). These separate VCF files were then integrated using bcftools-1.9, as recommended by the ICGC data coordination center (DCC) [13]. Germline variant calling was originally performed by the ICGC working group [12]. This group used six different variant callers: GATK HaplotypeCaller, FreeBayes, Real Time Genomics (RTG), Delly, TraFiC mobile element insertion caller (https://gitlab.com/mobilegenomes/TraFiC), and Eagle2 to assemble the SNVs, indels, and structural variations (SV). The final set of PCAWG germline calls comprises 2,642 donors who are composed of four subpopulations as described in Additional file 1: Table S1 European/American (1,904 donors; 72.1%), East Asian (396 donors; 15.0%), African (125 donors; 4.7%), and South Asian (36 donors; 1.4%). This also contains 181 donors (6.9%) whose ethnic information was not available.

### Control exomes from the 1000 Genomes Project

Exome sequences of healthy controls were collected from the 1000 Genomes Project (1 KG; Phase III high-coverage whole-exome sequences) [14] VCF from the FTP server (ftp://ftp.1000genomes.ebi.ac.uk/vol1/ftp/release/20130502/) under the following authorization (dbGaP phs000710.v1.p1). The data covers 2,504 individuals from four subpopulations (European, *N* = 503; American, *N* = 347; African, *N* = 661; East Asian, *N* = 504; South Asian, *N* = 489, Additional file 2: Fig. S2a). Data generation and variant calling procedures are detailed in the 1 KG flagship paper [14].

### Clinical information

Clinical and histological annotation for PCAWG donors were obtained from the ICGC Portal [12] (https://dcc.icgc.org/releases/PCAWG/clinical_and_histology). PCAWG data included 1,462 males (55.3%) and 1,180 females (44.7%). The Pan-cancer cohort comprises 37 distinct tumor types (Additional file 1: Table S1). Cancer patients’ normal samples were mainly derived from blood and non-tumor tissues adjacent to the primary stie or other sites such as bone marrow and lymph node. The ethnicity and gender annotation for 1 KG samples is available from the 1000 Genome portal (https://www.internationalgenome.org/data). No additional phenotype information was collected since 1 KG is a freely available genomic data set.

### Variant annotation and filtering step

To generate a single-standard functional annotation pipeline, we compiled each VCF file from PCAWG (*N* = 2,642) and 1 KG (*N* = 2,504). First, we discarded variants in ENCODE/DUKE [15] and DAC blacklist [16] regions to filter out variants in low mappability regions and we selected variants only in the ENCODE/CRG GEM mappability region (75mers) [17]. Next, we annotated the collected variants in the VCF file using Annovar (version 2014 Apr 14) [18]. Of the data Annovar reports, we used (i) the clinical and phenotypic effect of variants via ClinVar [19] (accessed on 18 June 2019), (ii) minor allele frequencies (MAFs) of variants across eight subpopulations: African/African American (AFR), South Asian (SAS), East Asian (EAS), Latino/Admixed American (AMR), non-Finnish European (NFE), Finnish European (FIN), Ashkenazi Jewish (ASJ) and Other (OTH) and also globally in The Genome Aggregation Database (gnomAD) exome (v2.1.1) [20]. Simultaneously, we also used Variant Effect Predictor (VEP)-release-96 [21] to provide the gene-based information of canonical transcripts. Of the data VEP reports, we used the consequences of the protein-coding variants: synonymous, missense, stop gain and loss, splice site, frameshift insertion/deletion (indel), and in-frame indel. Next, we discarded all variants marked as potential technical artifacts in the gnomAD exome including excess heterozygosity at a variant site (InbreedingCoeff), allele count zero variants after filtering out low-confidence genotypes (AC0) and failed random forest filtering threshold (RF). The differences in the number of detected variants between the two groups were not observed across three types of variants (median number of variants per sample in PCAWG had 17,320 of total variant, 7,788 missense, and 364 PTVs, while 17,464 total, 7,843 missense and 369 PTVs in 1 KG; Additional file 2: Fig. S1; Pearson correlation coefficient between PCAWG and control for three types of variants = 0.96, respectively).

### Principal component analysis using common variants

We expect many germline variants from whole-exome sequencing data or genome-wide association studies to vary according to the different ethnicities within the cohort. To account for the potential confounding effects from population stratifications, we conducted a principal component analysis (PCA) using only the common germline variants (non-synonymous) with ≥ 5% MAF, both globally and within each ethnic group in the gnomAD exome and 90% genotyping rate using PLINK [22] version 2.0. We performed two separate PCAs: (i) a case–control analysis using PCAWG and 1 KG data, and (ii) a two-hit preference analysis using only PCAWG data (Additional file 2: Fig. S2).

### Rare pathogenic variants

We defined rare variants as those with a frequency < 0.5% globally and within each of the eight subpopulations in the gnomAD exome. We confirmed the distribution of MAFs of clinically validated pathogenic variants. The excess of pathogenic variants in cancer was robust when applying a 0.1% MAF globally and within each of the eight subpopulations in the gnomAD exome (Additional file 2: Fig. S11). For variants not present in the gnomAD exome, we checked the detection frequency of variants in our cohorts (PCAWG, 1 KG separately) to avoid including TCGA or 1 KG biased variants, and discarded variants detected in more than 1% of samples in either the PCAWG or 1 KG cohort.

From the collected rare variants, we defined pathogenic variants using two conditions: (1) Protein truncation variants (PTVs; as potentially deleterious variants) were included splice site, frameshift indel, and stop gain/lost variants as annotated by VEP (release-96) [21]. Additionally, we included predicted splicing donor or acceptor loss variants with a score greater than 0.8 by SpliceAI (https://github.com/Ensembl/VEP_plugins/blob/release/109/SpliceAI.pm). To collect only highly confident PTVs, we applied several filtering steps: (i) removing PTVs annotated as “Benign” or “Likely benign” in ClinVar [19], and (ii) excluding PTVs located in either terminal exon or without functional domains (https://www.ebi.ac.uk/interpro/). (2) ClinVar pathogenic variants were defined with ‘Pathogenic’, ‘Likely pathogenic’, ‘association’, and ‘risk factors’ with related clinical evidence developed by the American College of Medical Genetics and Genomics (ACMG) in ClinVar [19].

### Gene set classification

We retrieved the genes with known to cause in clinical diseases and phenotypes from the Online Mendelian Inheritance in Man (OMIM) databases [8] (https://www.omim.org/downloads; Jan 21, 2020) via the Gene Map (cytogenetic locations of genes; *genemap2.txt*) and Morbid Map (cytogenetic locations of disorders; *mim2gene.txt*). The OMIM database provides 17,076 genes linked to 5,392 diseases represented in the Gene Map and Morbid Map. We selected 5,460 disease-associated genes, each associated with at least one phenotype (genetic disease), from *genemap2.txt* for further analysis. Using the R package *annotables* (https://github.com/stephenturner/annotables), we annotated these genes to their Ensemble gene names, resulting in 4,095 unique genes. Furthermore, we collected a total of 152 known CPGs from recent studies [23] including 114 genes from a recently published review paper [4], 11 genes from Cancer Gene Census-Germline (http://cancer.sanger.ac.uk/census/), 12 genes from references search (details in from Huang et al. [23]), and 15 genes from the St. Jude PCGP germline study [24].

To compile a list of highly confident somatic driver genes, we first collected 1,196 genes across four data sources: IntOGen [25], MutSig [26], MutPan [27], and Cancer Gene Census-Somatic [28]. To avoid overlapping gene sets, we first defined the CPGs (*N* = 152), then identified 3,952 OMIM genes, excluding 143 that overlapped with CPGs. Next, we defined 697 SODs after removing 499 genes that overlapped with either CPGs or OMIM genes. Subsequently, we excluded 53 CPGs from the final CPG set, which had no clinically proven pathogenic variants from ClinVar found in cancer consortium data sets (either TCGA or PCAWG). We considered only those genes with at least three pathogenic variant carriers in a pan-cancer study to increase statistical power. Ultimately, we defined 1,224 genes as input genes, including 36 CPGs, 116 SODs, and 1,072 OMIM genes, which comprised 658 autosomal-recessive [AR] genes, 229 autosomal-dominant [AD] genes, 85 AD-AR genes (i.e., variants from the same gene having dual effects, either dominant or recessive), 5 X-linked genes, 7 somatic, 3 digenic, 2 isolated, 3 multifactorial, and 80 genes with unknown inheritable phenotypes, based on distinctive inheritance annotation (Additional file 1: Table S2).

### Statistical analysis of pathogenic variant enrichment

We performed a burden test to compare the enrichment of pathogenic variants within genes between cancer cases (PCAWG) and controls (1 KG). This method aims to measure gene-level cancer risk enrichment by consolidating potentially rare pathogenic variants using a generalized linear regression model (GLM) via a stats package in R. We implemented this model on each gene in a pan-cancer study as follows:

$$glm\ (N \sim Germline\ Variants + PC1 + PC2 + PC3 + PC4,\;family= ``binomial")$$

**Fig. 2:**
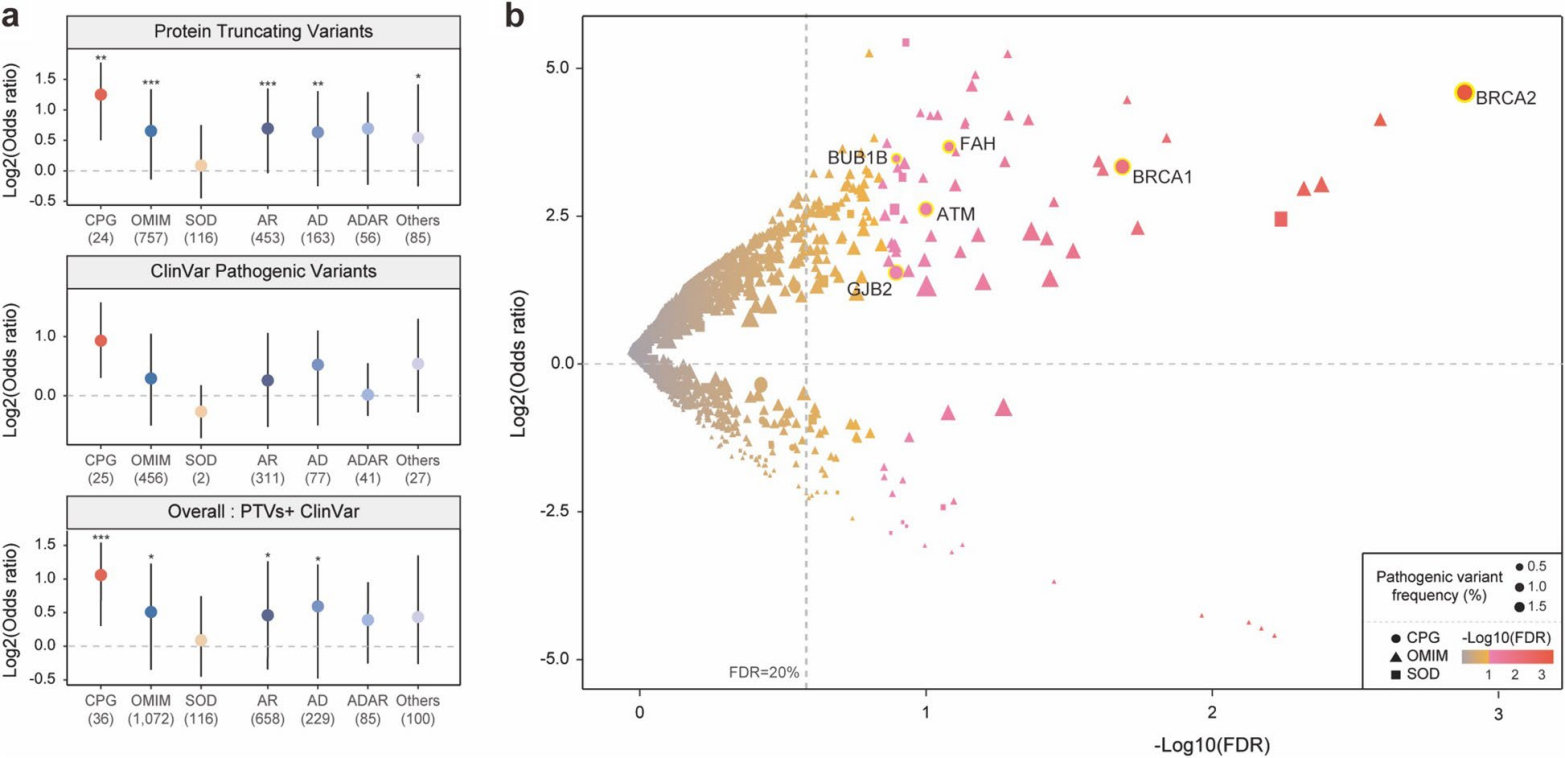
Enrichment of pathogenic variants in 2,642 cases compared to 2,504 control samples. **a** Enrichment of pathogenic variants in cases compared to control samples for three gene sets and four OMIM subgroups (* *P* < 0.05, ** *P* < 0.01, *** *P* < 0.001). The median value of each gene set is displayed as a circle. The length of each whisker represents 1.5 times the interquartile range (shown as the height of each box). **b** Excess of pathogenic variants (PTVs and ClinVar pathogenic variants) in case samples compared to control samples for 1,265 individual genes. The dashed horizontal line indicates a Log2(odds ratio) equal to zero, and the dashed vertical line represents the statistical significance threshold (*FDR* = 20%). Color indicates significance, shape represents the type of gene, and size presents the frequency of pathogenic variants. Significantly enriched CPGs are highlighted with a yellow boundary

Here, *N* = case (1) or control (0), Germline Variants = the number of samples carrying rare pathogenic variants for each gene-tissue pair, and PC1-PC4 are principal component values derived from PCA analysis of PCAWG and 1 KG data, utilized to control population structures. We applied PTVs or ClinVar pathogenic variants separately and together (Fig. 2a). We computed the regression coefficient and *P*-value for individual gene-tissue pairs using the summary function in R. More positive coefficient values signify stronger enrichment of pathogenic variants in the case compared to the control. Additionally, we examined if including gender as a factor would influence the regression results. We confirmed that the model's output remains consistent, regardless of the presence or absence of the gender effect (see Additional file 2: Fig. S3). For single-cancer type analysis, we only considered the single, most prominent population due to the statistical issue of vastly biased population discrepancies with a small sample size compared to pan-cancer.

### Pathogenic variant enrichment in disease class and pathways

To systematically estimate the enrichment of pathogenic variants in disease classes compared to controls, we compiled 10 diseases classes (Cardiovascular, Endocrine, Epilepsy, Hematology, Immune, Liver, Lung, Metabolism, Muscular/Skeletal, and Neurological) encompassing 96 hereditary diseases based on clinically relevant disease diagnosis from Centogene (https://www.centogene.com/diagnostics/ngspanels.html) and Blueprint Genetics (https://blueprintgenetics.com/tests/panels/).

To investigate the biological knowledge associated with the 109 genes and their pathogenic variants, we first conducted a web-based functional annotation analysis using the Database for Annotation, Visualization and Integrated Discovery (DAVID; https://david.ncifcrf.gov/) [29]. Using DAVID, we mapped gene-level functional annotations (GO) and signaling pathway information (KEGG) to individual genes (see Additional file 1: Table S5). Next, we performed an enrichment analysis for each disease or gene ontology/pathway in a pan-cancer study as follows:

$$glm\ (N \sim Pathway\ or\ Disease\ classes + PC1 + PC2 + PC3 + PC4, family= ``binomial")$$

where: *N* = case (1) or control (0), *Pathway or Disease* = the number of samples carrying a rare pathogenic variant for each disease or pathway-related gene. PC values from PCA analysis of PCAWG and 1 KG served as input for the regression model.

### Loss-of-heterozygosity event from PCAWG

We retrieved genomic copy-number alteration (CNA) data, which includes loss-of-heterozygosity (LOH) information, from the International Cancer Genome Consortium (ICGC) data portal [30, 31]. This dataset provides gene-level calls across 2,642 samples and can be accessed at ICGC (https://dcc.icgc.org/releases/PCAWG/consensus_cnv/gene_level_calls). It encompasses summarized intersections of genomic region calls from six CNA callers: ABSOLUTE, ACEseq, Battenberg, cloneHD, JaBbA, and Sclust, as detailed in Dentro et al. [32]. LOH was determined from the CNA data (all_samples.consensus_CN.minor_allele.by_gene.170313.txt; Additional file 1: Table S6) using the profile of the minor copy number, defined as the least amplified allele, following the methodology described in the PCAWG paper [31]. Additionally, we compared our LOH classifications (either LOH or no-LOH) against observed copy number changes (either loss or gain) from the dataset (all_samples.consensus_level_calls.by_gene.170214.txt) for all examined genes. Our detected LOH events were categorized as losses (77.2%), wild-type (18.9%), or gains (3.4%). In comparison, the proportions for no-LOH events were 5.2%, 83.1%, and 11.8%, respectively (Additional file 2: Fig. S8).

### Statistical analysis of two-hit preference

To test the two-hit hypothesis, we performed an analysis to check for an excess of pathogenic variants within a gene in samples with LOH events compared to samples without such events across cancer types. We applied the model to each gene-tissue pair as follows:

$$glm\ (N \sim Germline\ Variants + PC1 + PC2 + PC3 + PC4, family = ``binomial")$$

In this model, *N* represents samples with LOH event (1) or samples without LOH event (0), *Germline Variants* indicates the number of samples carrying rare pathogenic variants (PTVs + ClinVar pathogenic variants) for each gene-tissue pair. The principal component values derived from PCA analysis, performed solely on cancer samples to control for population structures (Additional file 2: Fig. S2e), were used as an input for the regression. We computed the regression coefficient and *P*-value for each gene-tissue pairs using the *summary* function in R. More positive coefficient values indicate a stronger enrichment of pathogenic variants in samples with LOH compared to samples without LOH.

### Case–control analysis in a single cancer

For the analysis of individual cancer types, we focused only on the nine cancer types with a sample size greater than 100 to ensure robust analysis. These include Breast-AdenoCA (*N* = 194), CNS-Medullo (*N* = 145), Kidney-RCC (*N* = 143), Liver-HCC (*N* = 317), Lymph-BNHL (*N* = 107), Ovary-AdenoCA (*N* = 113), Panc-AdenoCA (*N* = 238), Prost-AdenoCA (*N* = 210), and Skin-Melanoma (*N* = 107). We then applied the regression model for pathogenic variant enrichment, disease/pathway analysis, and the two-hit preference analysis to the major ethnic group for each cancer type (East Asian for Liver-HCC and European for the remaining eight cancer types, as detailed in Additional file 1: Table S1). The threshold for the number of samples carrying a pathogenic variant in each cancer type was carefully adjusted to avoid inflation or depletion dependent on the cancer type. We included genes with more than two carriers either in cases (samples with LOH) or controls (samples without LOH) for CNS-Medullo and Liver-HCC, more than three for Kidney-RCC, Lymph-BNHL, Ovary-AdenoCA, Panc-AdenoCA, Prost-AdenoCA, and Skin-Melanoma, and more than four for Breast-AdenoCA (Additional file 2: Fig. S4).

### PCA for the gene classification

To classify significantly enriched genes (*N* = 109), we integrated three features: (i) Two-hit preferences in a pan-cancer (excess of pathogenic variants in samples with LOH event compared to samples without LOH event, as determined by the regression model; measured as a Log2 odds ratio), (ii) TAU score [33], which indicates gene expression patterns across various tissues from GTEx data [34] (from 0 to 1: close to 1 indicates a tissue-specifically expressed gene), (iii) The loss-of-function tolerance value, the loss of function observed/expected upper bound fraction (LOEUF). This scale measures genes predicted for loss-of-function (pLoF) constraint, as determined by gnomAD [20]. We used three values as input for PCA using the *prcomp* function in the R package stats and defined four clusters using the enhanced *K*-means clustering method in the R package factoextra package (https://cran.r-project.org/web/packages/factoextra/index.html) [35].

### Independent cancer cohort from TCGA and gnomAD

For independent validation, we applied two different databases: TCGA (as an independent case set; for TCGA vs. 1 KG) and gnomAD (as an independent control set; for PCAWG vs. gnomAD). Germline variants for 10,389 TCGA samples [23] were obtained from the National Cancer Institute (NCI) Genomic Data Commons (GDC) [36] legacy archive (dbGaP phs000178; PCA.r1.TCGAbarcode.merge.tnSwapCorrected.10389.vcf). After excluding 782 samples overlapping with the PCAWG study, we collected a final sample of 9,607 TCGA cases. This sample included four populations: European (*N* = 7,072; 73.6%), Asian (*N* = 603; 6.3%), African (*N* = 793; 8.3%), and other or unknown ethnicity (*N* = 1,139; 11.9%). We also used gnomAD v2 exome [20] as an independent control set, performing additional variant annotation steps with both Annovar and VEP to align with the annotation used in the PCAWG data. We extracted pathogenic variants in gnomAD using the same variant collection steps as previously described. To circumvent issues with population differences, our independent validation focused on the European population. Among the 109 CPG-like OMIM genes, pathogenic variants were also found in 93 genes within the European population. We calculated pathogenic variant frequencies across genes by summing all non-cancer European allele frequencies, since individual variant information is not available in gnomAD. Finally, we measured the differences between PCAWG_European and non-cancer European gnomAD data.

### Metabolome analysis of *PAH* carriers

To investigate metabolite changes due to PAH alteration, we collected blood samples from 8 PAH heterozygote carriers (parents of Phenylketonuria patients) and 22 healthy controls (without the phenylketonuria phenotype) at the Seoul National University Hospital, South Korea approved by the Seoul National University Hospital Review Boards (IRB No. H-2101–196-1193). Phenylketonuria (PKU) is caused by genetic variants in both alleles of the PAH gene (i.e., homozygotes). Therefore, we assumed that the parents of PKU patients would be heterozygote carriers. All carriers and controls were of Korean ethnicity. The metabolic changes associated with PAH heterozygote carriers were assessed at Human Metabolome Technologies, Inc. (HMT), with the samples being processed according to the HMT protocol (Document ID: BLB.1.0.0). Metabolic changes were evaluated using the Cation and Anion modes of CE-TOFMS (Capillary Electrophoresis Time-of-Flight Mass Spectrometry) for metabolome profiling. We extracted 220 annotated peaks from the CE-TOFMS analysis using automatic integration software (MasterHands ver.2 [37]), which included the mass-to-charge ratio (m/z), migration time (MT), and peak area. We used the following equation to convert the peak area to the relative peak area.

$$Relative\ Peak\ Area= \frac{Metabolite\ Peak\ Area}{Internal\ Standard\ Peak\ Area\ \times\ Sample\ Amount}$$

We then assigned putative metabolites from HMT's standard library and Known-Unknown peak library using the m/z and MT values. All metabolite concentrations were measured using standard curves derived from single-point (100 $\upmu$ M) calibrations, and the peak area of each metabolite was normalized to the area of the internal standard. We selected 110 metabolites with quantified concentrations for disease-related enrichment analysis (Additional file 2: Fig. S10c) by MetaboAnalyst 5.0 [38] (https://www.metaboanalyst.ca/), a web-based metabolome data analysis tool.

### Metabolic simulations using genome-scale metabolic models

We used a genome-scale metabolic model (GEM) [39] for metabolic simulations in PAH carriers and non-carriers within PCAWG. GEM is a computational model that incorporates information on the biochemical reactions of all metabolic genes in a cell, and it can be simulated using optimization techniques for various metabolic studies [40]. In this study, a previously developed generic human GEM Recon 2M.2 [39] was transformed into a context-specific (or patient-specific) GEM by integration it with RNA-Sequencing data from PCAWG-TCGA Liver-HCC and Lung-SCC samples. The Task-driven Integrative Network Inference for Tissues (tINIT) method, complemented by a rank-based weight function, was employed to generate patient-specific GEMs. We first predicted metabolites secreted from the PAH carriers (*N* = 3) and the non-carriers (*N* = 309) using the Liver-HCC GEMs. The Liver-HCC GEMs predicted PAH carriers and non-carriers to secrete 16 and 40 metabolites, respectively. The predicted metabolite secretion rates from the PAH carriers with Liver-HCC were then used as input for the GEMs representing the PAH carriers with Lung-SCC (*N* = 15). Similarly, the predicted metabolite secretion rates from the PAH non-carriers with Liver-HCC were used as input for the GEMs representing the PAH non-carriers with Lung-SCC (*N* = 478). To simulate the Lung-SCC GEMs, we arbitrarily limited the maximum uptake rate for the predicted metabolites from the Liver-HCC GEMs to 1.1 times the average metabolite secretion rates. Exchange reactions for inorganic nutrients were set to have the maximum secretion and uptake rates of 1,000 and -1,000 mmol/gDCW/h, respectively. We did not allow uptake of other nutrients when simulating the Lung-SCC GEMs. We predicted metabolic fluxes of the patient-specific GEMs using the least absolute deviation (LAD) method, which aims to minimize the distance between transcript expression levels from RNA-seq data and target reaction fluxes to be calculated. Both the tINIT with the rank-based weight function and the LAD method were implemented using in-house Python scripts [39].

### Functional analysis with *PAH*

We used 493 RNA sequencing (RNA-seq) data from the PCAWG-TCGA Lung-SCC via the GDC Data portal (https://portal.gdc.cancer.gov), for the PAH analysis. Gene expression values were calculated post-transcriptomic read alignment to the human reference genome (hg19) using spliced transcripts alignment to a reference (STAR) version 2.5.3a [41] and RSEM v1.3.0 (RNA-seq by Expectation Maximization) [42]. Gene set enrichment analysis (GSEA) was performed using the Java application version 4.1.0 [43] to evaluate the functional enrichment in Cancer Hallmark with 1,000 permutations. In addition, we calculated the transcriptional score (TS) [44], defined as an absolute sum of gene expression correlation coefficient values using 38 immune checkpoint modulatory genes [45]. We compared TS values among three groups of samples (1) *PAH* carrier in Lung-SCC, (2) *PAH* non-carrier in Lung-SCC, and (3) normal lung samples in GTEx [34]. Given the significantly smaller number of PAH carriers in Lung-SCC compared to either PAH non-carriers in Lung-SCC or GTEx normal lung samples, we randomly selected an equal number of samples 1,000 times (*N* = 45 samples of PAH non-carriers and GTEx normal lung group, separately). The mean TS values calculated from random sampling with non-PAH and GTEx samples were then compared with the observed TS value of PAH carriers.

### Korean Lung-SCC germline variant calling

For the independent validation of *PAH* pathogenic variant frequency in lung cancer, we collected 245 peripheral blood mononuclear cell (PBMC) specimens from Korean patients with lung squamous cell carcinoma. This was carried out with informed consent from Samsung Medical Center (SMC) under the IRB No. 2013–10-112 and 2008–06-033. The samples were processed using the Swift 2S Turbo DNA library kit, and whole exome sequencing (WES) data was generated with the IDT xGen Exome Research Panel v1.0 kit on the Illumina NovaSeq 6000 platform. The variant calling step was conducted using the Genome Analysis Toolkit (GATK) Best Practices Workflow pipeline, such as the HaplotypeCaller [46]. We followed the same variant annotation process as described above.

## Results

### A framework for measuring enrichment of germline variants in cancer

To estimate the contribution of pathogenic variants in OMIM genes to cancer systematically, we developed a statistical method that tests the excess of pathogenic variants in cancer patients relative to healthy control samples.

We compared the frequency of pathogenic variants in 2,642 cancer patients from the PCAWG [12] study to 2,504 healthy control exomes from the 1 KG project [14] (Fig. 1b and Additional file 1: Table S1). Rigorous quality control steps were applied to the two distinct datasets, and we only considered variants in regions with sufficient and comparable sequencing coverage in both PCAWG and 1 KG samples (see Methods; Additional file 2: Fig. S1). Despite applying filtering steps to both datasets, some technical differences might remain due to the different variant-calling pipelines.

Germline variants were categorized as rare pathogenic variants if they exhibited a minor allele frequency (MAF) < 0.5% in the Genome Aggregation Database (gnomAD) exome [20] (see Methods; Fig. 1b). Subsequently, we gathered protein truncation variants (including splicing variants, frameshift indels, and nonsense variants; PTVs) and variants clinically validated in the ClinVar database [19] (ClinVar pathogenic). We also considered the overall PTVs and ClinVar pathogenic (incorporating either PTVs or ClinVar pathogenic) to estimate the maximum possible contribution of pathogenic variants to cancer (see Methods; Fig. 1b). We focused on three gene sets: (i) previously known cancer predisposition genes (CPGs) based on a recent study [23], (ii) OMIM genes, and (iii) somatic driver genes (SODs) that frequent somatic alterations are associated with an increased cancer risk in multiple databases. Ultimately, we identified 7,012 pathogenic variants (either PTVs or ClinVar pathogenic variants) in 2,534 genes that have at least one defined pathogenic variant. Among these variants, 77.8% (5,452 out of 7,012) were PTV variants, and 32.5% (2,280 out of 7,012) were clinically pathogenic as ClinVar pathogenic, including 10.3% overlap between PTVs and ClinVar pathogenic variants (*N* = 720).

We gathered single nucleotide variants (SNVs) and indels for each gene. The case–control analysis was designed to test for an excess of pathogenic variants in cancer patients (cases) compared to controls (non-cancer patients). This analysis utilized a burden test with population adjustment, which accounted for the first four principal component (PC) values (explaining > 90% of variances; Additional file 2: Fig. S2a-b) in order to control for population structure. This structure was estimated from common variants of case–control samples (see Methods; Fig. 1b and Additional file 2: Fig. S2c-d). We set a threshold to define the genes to be tested based on the total number of carriers with pathogenic variants in both case and control samples. This was done to ensure there was no inflation in the distribution of observed *P*-values (Additional file 2: Fig. S4). Consequently, the genes to be tested were defined as those carrying at least three pathogenic variants (either PTVs or ClinVar pathogenic) in the pan-cancer (all cancer samples together) case–control analysis.

### Enrichment of pathogenic OMIM gene variants genes across cancers

In our pan-cancer analysis, we found that cancer predisposition genes (CPGs) showed significant enrichment of PTVs in cases (median Log2 odds ratio (OR) for the enrichment of PTVs in cases = 1.25, *P* = 1.07 × 10^−3^ by Wilcoxon rank sum test, Fig. 2a and Additional file 1: Table S2). The distribution of ORs (without logarithmic transformation) is also presented in the Additional file 2: Fig. S5. Notably, we also observed that OMIM genes exhibited significant enrichment for PTVs in cases compared to controls (median Log2OR = 0.65, *P* = 1.54 × 10^−4^ by Wilcoxon rank sum test, Fig. 2a). This enrichment was also consistent or stronger when considering OMIM sub-groups separately, OMIM-Autosomal Recessive genes (OMIM-AR; median Log2OR = 0.69, *P* = 4.08 × 10^−5^ by Wilcoxon rank sum test) and Autosomal Dominant genes (OMIM-AD; median Log2OR = 0.63, *P* = 7.62 × 10^−3^ by Wilcoxon rank sum test). Somatic driver genes (SODs; *N* = 116) did not show significant enrichment in cases compared to controls (median Log2OR = 0.09), indicating that rare protein truncating variants (PTVs) from OMIM genes could contribute to an increased cancer risk.

Considering ClinVar pathogenic variants, we observed marginal enrichment of pathogenic variants in CPGs in the pan-cancer case–control analysis (*N* = 25; median Log2 OR = 0.99; *P* = 2.39 × 10^−1^ by Wilcoxon rank sum test, Fig. 2a and Additional file 1: Table S2). This lower enrichment might be due to the smaller number of ClinVar pathogenic variants (which are clinically validated variants; 5,452 PTVs vs. 2,280 ClinVar pathogenic variants) compared to PTVs. OMIM genes (*N* = 456) also showed marginal enrichment (median Log2 OR = 0.31;* P* = 4.52 × 10^−1^ by Wilcoxon rank sum test) and only two ClinVar pathogenic variants were detected in SODs (median Log2 OR = -0.29), as expected based on their definition (cancer genes discovered by recurrent somatic alterations but not inherited variants).

When analyzing both PTVs and ClinVar pathogenic variants together in CPGs (*N* = 36), we saw robust enrichment in the pan-cancer dataset (median Log2 OR = 1.06; *P* = 4.66 × 10^−4^ by Wilcoxon rank sum test; Fig. 2a). We also found that pathogenic variants in OMIM genes consistently show significant enrichment in cancer compared to control (*N* = 1,072; median Log2 OR = 0.51; *P* = 1.53 × 10^−2^ by Wilcoxon rank sum test). This included OMIM-AR (median Log2 OR = 0.46; *P* = 1.18 × 10^−2^ by Wilcoxon rank sum test) and OMIM-AD (median Log2 OR = 0.60; *P* = 4.67 × 10^−2^ by Wilcoxon rank sum test). In contrast, SODs (*N* = 116) did not show any difference in pathogenic variant enrichment between cancer patients and the controls, consistent with the analysis of PTVs and ClinVar pathogenic variants (median Log2 OR = 0.09). To ensure the analysis of as many potential pathogenic variants as possible, from this point forward, our definition of rare pathogenic variants will encompass both PTVs and clinically validated variants.

We identified 109 genes (6 CPGs, 103 OMIM genes) that individually showed an enrichment for pathogenic variants (PTVs + ClinVar pathogenic variants) in pan-cancer at a false discovery rate (FDR) of less than 20% (Fig. 2b and Additional file 1: Table S2). These data included six previously known CPGs: *BRCA2* (Log2 OR = 4.21, *FDR* = 4.51 × 10^−4^), *BRCA1* (Log2 OR = 3.01, *FDR* = 1.07 × 10^−2^), *FAH* (Log2 OR = 3.33, *FDR* = 5.33 × 10^−2^), *ATM* (Log2 OR = 2.32, *FDR* = 6.60 × 10^−2^), *BUB1B* (Log2 OR = 3.14, *FDR* = 8.69 × 10^−2^), and *GJB2* (Log2 OR = 1.28, *FDR* = 8.71 × 10^−2^). Their targeting cancer types were presented in Additional file 1: Table S3. Pathogenic variants of 103 OMIM genes (10.2% of tested OMIM genes) were found to be significantly enriched in cancer patients. This includes 63 OMIM-ARs, 24 ADs, 3 ADARs, and 13 Others. For example, *PRSS12* displayed the highest significant enrichment of pathogenic variants in cancer compared to control (Log2 OR = 3.74, *FDR* = 9.86 × 10^−4^), next to *OTOG* (Log2 OR = 2.68, *FDR* = 1.70 × 10^−3^), *ATP7B* (Log2 OR = 2.61, *FDR* = 2.00 × 10^−3^), *AAGAB* (Log2 OR = 3.45, *P* = 7.12 × 10^−3^), and *PAX4* (Log2 OR = 1.98, *FDR* = 9.31 × 10^−3^). Hereafter, we will refer to these 109 genes as CPG-like OMIM genes (including 6 CPGs). They show significant enrichment of pathogenic variants in cancers, as determined by using PTVs and ClinVar pathogenic variants in the pan-cancer analysis at an FDR of 20% (Fig. 2b).

Furthermore, we confirmed that pathogenic variants show specific enrichment in CPG-like OMIM genes by examining independent data sets for cancer data and control sets. For the independent cancer set, The Cancer Genome Atlas (TCGA) was used, which covers 10,389 cancer patients across 33 cancer types, providing a comprehensive independent cancer data set [23]. In addition, we used gnomAD [20], currently the largest collection of human sequencing data, as a control data set after removing allele frequencies from cancer samples (i.e., gnomAD-nonCancer). For validation of our case–control analysis, we focused on a European population to reduce ethnically biased sequence variability in both the case and control datasets. By applying two combinations of case–control analysis (TCGA vs. 1 KG and PCAWG vs. gnomAD), we observed robust enrichment of pathogenic variants in CPG-like OMIM genes in cancer cases compared to controls. We confirmed that 52.7% to 95.7% of CPG-like OMIM genes presented enriched pathogenic variants in cancer compared to control (Additional file 2: Fig. S6).

### Enrichment of rare germline variants of OMIM genes in specific cancers

Next, we applied a case–control analysis to the 9 cancer types with more than 100 samples (comprising 60% of the samples in a pan-cancer; Additional file 1: Table S1). To test for enrichment in single-cancer types versus controls, we selected genes based on the total number of pathogenic variant carriers from cases and control. This helped avoid inflation or depletion in each cancer type separately (Additional file 2: Fig. S4). In total, 246 genes (15 CPGs and 231 OMIM genes with 394 gene-cancer associations) were significantly associated with at least one individual cancer type. Of these, 41 genes were also significantly enriched in the pan-cancer dataset (Odds ratio = 2.67, Fisher’s exact test *P* = 8.15 × 10^−6^ at *FDR* 20%; Additional file 1: Table S4). Among the CPGs, *BRCA1* was significantly enriched in ovarian adenocarcinoma (Ovary-AdenoCA; Log2OR = 6.91, *FDR* = 9.63 × 10^−6^) and breast adenocarcinoma (Breast-AdenoCA; Log2OR = 4.81, *FDR* = 4.76 × 10^−3^) and *BRCA2* was enriched in Breast-AdenoCA (Log2OR = 4.70, *FDR* = 6.93 × 10^−5^), pancreatic adenocarcinoma (Panc-AdenoCA; Log2OR = 4.12, *FDR* = 6.45 × 10^−4^) and Ovary-AdenoCA (Log2OR = 3.76, *FDR* = 9.03 × 10^−3^). From OMIM genes, the top-ranked gene-cancer association was *EEF2* (Log2OR = 5.38, *FDR* = 1.40 × 10^−3^) with renal cell carcinoma (Kidney-RCC). Additionally, variants of *PEX1* (Log2OR = 5.13, *FDR* = 3.07 × 10^−3^) were enriched in B-cell non-Hodgkin’s lymphoma (Lymph-BNHL), *FLG2* (Log2OR = 5.04, *FDR* = 3.68 × 10^−3^) in Kidney-RCC, *DHCR7* (Log2OR = 4.81, *FDR* = 4.76 × 10^−3^) in Breast-AdenoCA and *FAM111A* (Log2OR = 4.68, *FDR* = 7.70 × 10^−3^) in medulloblastoma (CNS-Medullo) also showed significant enrichment.

### Functional annotation and disease classes represented in CPG-like OMIM genes

**Fig. 3:**
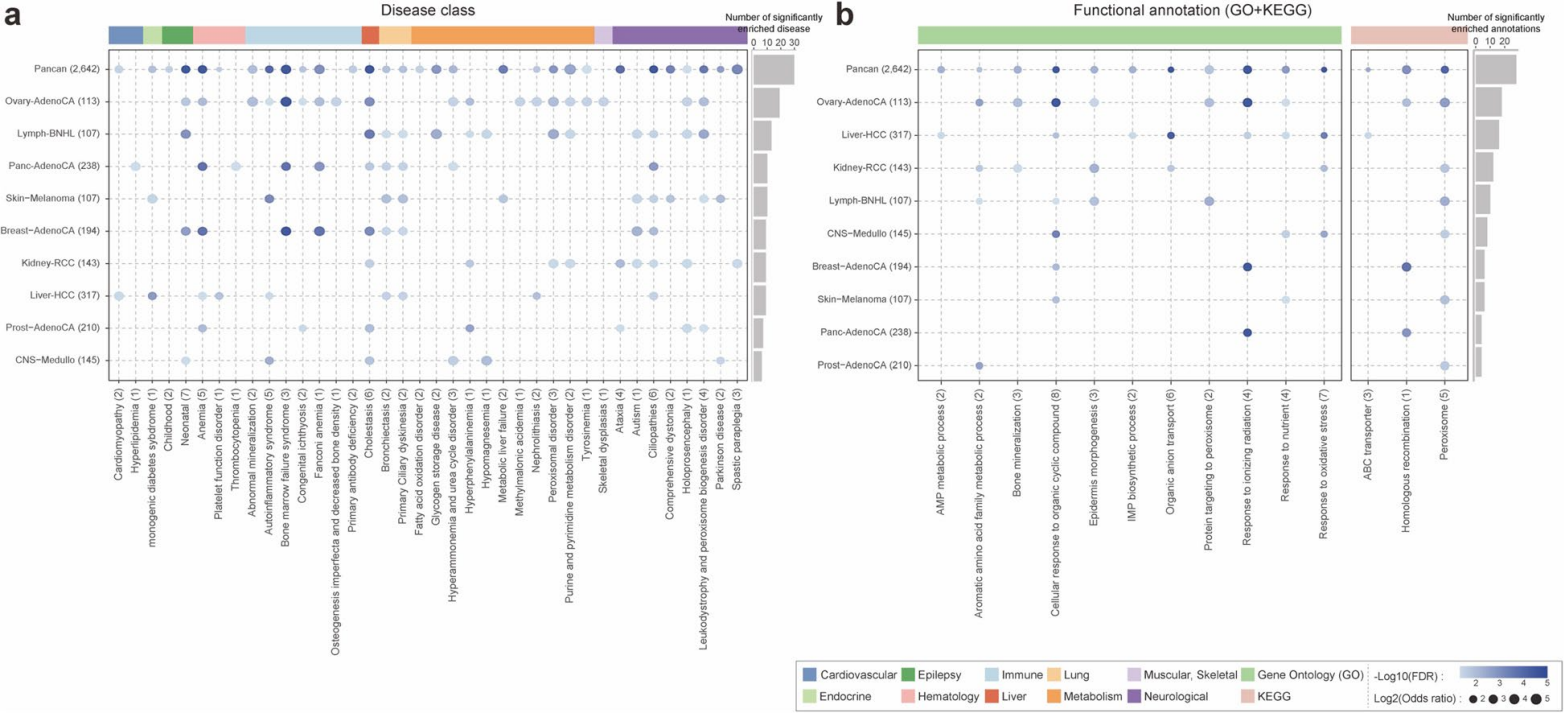
Pathogenic variants enriched in (**a**) diseases and (**b**) functional clusters across pan-cancer and single-cancer types using the linear regression model. Circle size indicates the excess of pathogenic variants per gene set, and color represents the significance of the enrichment in a specific cancer type compared to the control (1KG). Numbers in parentheses indicate the number of samples in each cancer type (left) and the number of tested genes in each disease (or functional annotation and pathway; bottom). The bar plot on the right shows the number of detected diseases (or functional terms and pathway) in each cancer type at a false discovery rate (FDR) of less than 0.05

OMIM genes that cause different genetic diseases can also be grouped into disease classes based on the physiological system affected. To complement single-gene-based case–control analysis, we measured the enrichment of pathogenic variants in 49 genetic diseases assigned to 10 representative disease classes using a regression model (see Methods). From 49 genetic diseases with at least one pathogenic variant from CPG-like OMIM genes (*N* = 109), 38 diseases showed significant enrichment in at least one cancer type or pan-cancer compared to the control (77.6% at *FDR* < 5%; Fig. 3a). The results remain consistent when we incorporate an additional set of disease genes from the Clinical Genome Resource, which includes 463 newly identified disease-associated genes for 59 genetic diseases (43 out of 59 diseases, 72.9% at FDR < 5%; see Additional file 2: Fig. S7), including four new disease classes (Aminoacidopathy, RASopathy, Intellectual disability, and Motile ciliopathy). The metabolic disease class had the highest frequency of significantly enriched pathogenic OMIM variants (22.4%; 11 diseases). The neurological disease class had the second-highest frequency (16.3%; 8 diseases), followed by the immune disease class (14.3%; 7 diseases).

Examining this in more detail, glycogen storage disease from the metabolic disease class in Lymph-BNHL showed the strongest enrichment (Log2OR = 5.13, *FDR* = 2.61 × 10^−3^). This is consistent with previous data showing that glycogen metabolism is associated with an increased risk of malignancy and tumorigenesis [47, 48]. Moreover, the next most significant enrichments highlighted the following associations: peroxisomal disorder to Lymph-BNHL (Log2OR = 5.13, *FDR* = 2.61 × 10^−3^) and Ovary-AdenoCA (Log2OR = 4.76, *FDR* = 7.33 × 10^−3^), bone marrow failure syndrome to Ovary-AdenoCA (Log2OR = 4.83, *FDR* = 6.53 × 10^−10^), autism to Breast-AdenoCA (Log2OR = 4.81, *FDR* = 3.88 × 10^−3^), abnormal mineralization to Ovary-AdenoCA (Log2OR = 4.76, *FDR* = 7.33 × 10^−3^) and Fanconi anemia to Breast-AdenoCA (Log2OR = 4.70, *FDR* = 6.16 × 10^−5^). Interestingly, the comorbidity relationship between Fanconi anemia and breast cancer-associated pathway has previously been described [49, 50].

We also performed a deeper enrichment analysis of functional annotation using biological processes in gene ontology (GO) [51] and KEGG signaling pathways [52] (see Methods; Fig. 3b). Pathogenic variants in CPG-like OMIM genes were significantly enriched for 11 GO terms and 3 KEGG signaling pathways in at least one individual cancer type or a pan-cancer dataset compared to the control (FDR < 0.05). Overall, peroxisome-related pathways showed the strongest enrichment, including the peroxisome pathway from KEGG in Ovary-AdenoCA (Log2OR = 5.53, *FDR* = 9.42 × 10^−4^) and Lymph-BNHL (Log2OR = 5.13, *FDR* = 2.89 × 10^−3^) and protein targeting to peroxisome from GO in Lymph-BNHL (Log2OR = 5.13, *FDR* = 3.03 × 10^−3^). As expected, the classical cancer predisposition genes-associated pathway, homologous recombination showed significant enrichment in pan-cancer (Log2 OR = 4.21, *FDR* = 4.45 × 10^−4^), Ovary-AdenoCA (Log2 OR = 3.76, *FDR* = 7.25 × 10^−3^), Breast-AdenoCA (Log2 OR = 4.70, *FDR* = 6.42 × 10^−5^), and Panc-AdenoCA (Log2 OR = 4.12, *FDR* = 5.89 × 10^−4^). Moreover, epidermis morphogenesis in Kidney-RCC (Log2OR = 5.53, *FDR* = 9.42 × 10^−4^), response to ionizing radiation (Log2OR = 4.83, *FDR* = 6.53 × 10^−10^), and bone mineralization (Log2OR = 4.76, *FDR* = 7.58 × 10^−3^) in Ovary-AdenoCA also showed significant enrichment.

### Four possible classes in OMIM genes

**Fig. 4:**
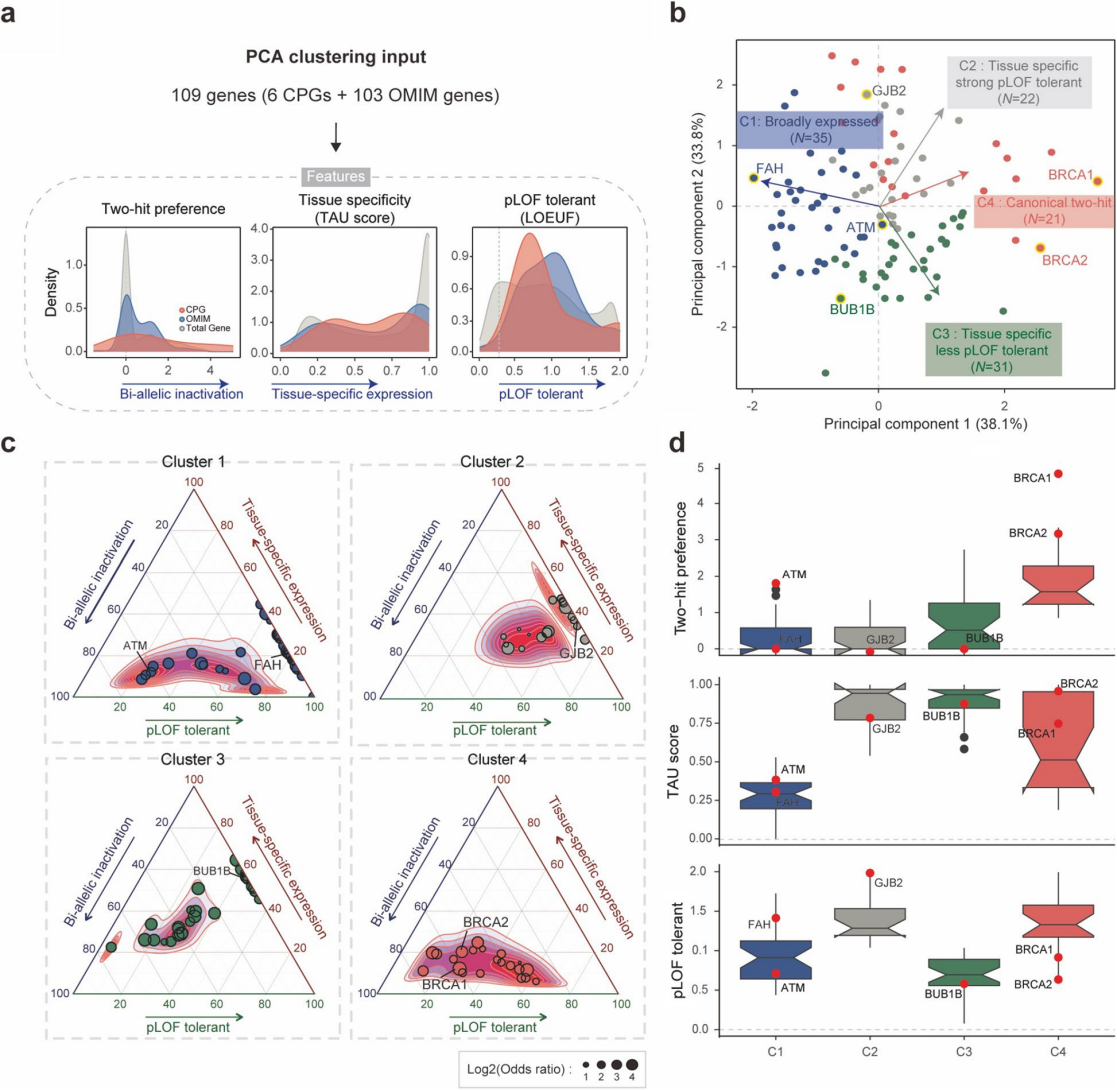
Four classes from 109 genes. **a** Schematic representation of gene classification by integrating three features: (i) two-hit preference: the excess of pathogenic variants in samples with LOH compared to samples without LOH (a more positive value indicates stronger two-hit preferences), (ii) TAU score: indicating the gene expression patterns across multiple tissues (from 0 to 1, close to 1 indicates tissue-specifically expressed gene), (iii) LOEUF: the scale of predicted loss-of-function (pLoF) constraint (a higher value indicates a pLOF tolerant gene). A LEOUF score below 0.35 indicates pLOF intolerance. **b** Principal components analysis with three features for 109 genes. Each color represents different clusters. **c** Ternary plot showing the distribution of the three features across four classes. CPGs are highlighted in each plot. The size presents the excess of pathogenic variants in cases compared to control samples (Odds ratio). **d** Box plot presenting the distribution of assigned values for each feature across clusters. CPGs are depicted as red dots with the name of each cluster. The black dots indicate outliers, which are values between 1.5 and 3 times the interquartile ranges

Many canonical CPGs are known to be constitutively expressed in tissues connected to their general biological function [4]. Moreover, they often adhere to the ‘two-hit’ hypothesis, where a pathogenic variant occurs in one allele of the gene and a ‘second hit’ occurs somatically in another allele, leading to an increased risk of cancer [4, 23]. However, exceptions to this broad tissue expression have been observed in several CPGs with tissue-specific functional roles [4], and diverse mechanisms that extend beyond the canonical CPG model are being proposed [6, 53]. Furthermore, it is been suggested that a single copy of an inherited CPG variant could be sufficient to increase cancer risk [6]. We hypothesized that pathogenic variant carriers might have an increased cancer risk through a variety of mechanisms beyond the classical understanding of CPGs. To systematically investigate how CPG-like OMIM genes contribute to tumorigenesis in diverse ways, we considered three defining features of the classes: (i) two-hit preference, (ii) tissue-specificity (TAU score), and (iii) loss-of-function intolerant value (LOEUF; Fig. 4a).

First, we applied Knudson’s two-hit hypothesis to the genes in the pan-cancer analysis. The two-hit preference was designed to measure the enrichment of pathogenic variants (PTVs and ClinVar pathogenic variants) in samples with loss-of-heterozygosity (LOH) compared to samples without LOH within a gene (see Methods; Additional file 2: Fig. S8). We again used a regression model adding four PCs to control for population structure, estimated from common variants from cancer samples only (Additional file 2: Fig. S2e). Next, we considered the gene expression pattern to reflect whether the gene is involved in a fundamental function or tissue-specific function [54]. We applied a TAU score, indicating the gene expression pattern from 27 tissues from GTEx [34], ranging from 0 (broadly expressed) to 1 (tissue specifically expressed). Lastly, LOEUF from gnomAD [20] which describes the tolerance to inactivation for predicted loss-of-function (pLoF) was used (genes below 0.35 LOEUF is considered as intolerant gene) [20].

We performed a principal component analysis (PCA) using the three features mentioned above as inputs for the genes. We defined four clusters using an enhanced *k*-means clustering algorithm (Additional file 2: Fig. S9a), suggesting that diverse mechanisms predispose OMIM genes to act like CPGs (Fig. 4b-c). The first cluster showed both low two-hit prevalence in a pan-cancer analysis and the lowest TAU score distribution, indicating that these genes are broadly expressed across tissues (Fig. 4d and Additional file 2: Fig. S9b). We, therefore, named them the ‘broadly expressed’ group. This cluster includes two CPGs (*ATM* and *FAH*) and 33 OMIM genes, including *ABCA7*, *CYP1B1*, *LRRK2*, *MVK*, *MMAA*, and *COL18A1* (Additional file 1: Table S7). The second cluster, including one CPG (*GJB2*), and 21 OMIM genes, is characterized by high TAU score and LOEUF scores but relatively low two-hit preferences in a pan-cancer analysis compared to single cancer types (Fig. 4d, Additional file 2: Fig. S9b-c). We refer to this cluster as the ‘Tissue-specific strong pLOF tolerant’ group of CPG-like OMIM genes. For example, *PRSS12* in Panc-AdenoCA, *ABCC11* in liver hepatocellular carcinoma (Liver-HCC), and *NOD2* in skin melanomas (Skin-Melanoma) show tissue-specific expression patterns (Additional file 1: Table 7 and Additional file 2: Fig. S9c). Genes in the third cluster have a high TAU score like the second cluster but have the lowest LOEUF values. This cluster includes one CPG (*BUB1B*) and 30 OMIM genes and is called the ‘Tissue-specific less pLOF tolerant’ group (Fig. 4d). The last cluster presents strong ‘two-hit’ preferences in a pan-cancer analysis as well as in single cancer types (Fig. 4d and Additional file 2: Fig. S9b-c) and includes two classical CPGs (*BRCA1* and *BRCA2)* and 19 OMIM genes. We therefore named this cluster the ‘Canonical two-hit’ group.

### *PAH* is a potential cancer-predisposing gene

**Fig. 5:**
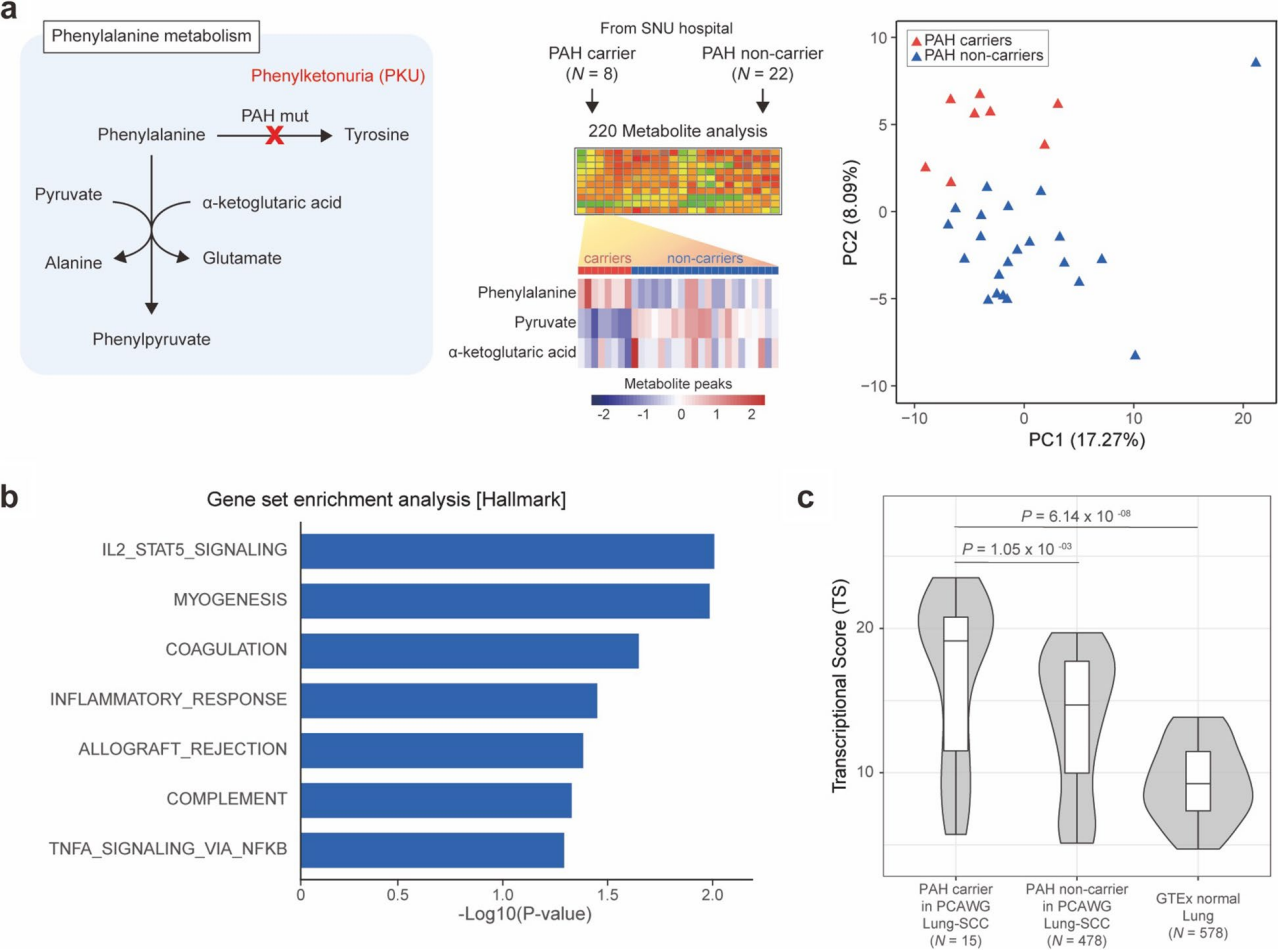
Contribution of *PAH* variants to the metabolic process. **a** Diagram of the phenylalanine hydroxylase (*PAH*) pathway and profiling of 220 metabolites for the 8 individuals heterozygous for pathogenic *PKU* variants and 22 healthy controls. In phenylalanine metabolism, L-phenylpyruvate can be generated from L-phenylalanine through two enzymatic reactions. The heatmap presents three representative metabolites (red: higher metabolite peaks in *PAH* carriers, blue: lower metabolite peaks in *PAH* carriers). PCA using the 220 metabolites clearly distinguished the *PAH* carriers from the *PAH* non-carriers. **b** Hallmark GSEA signatures from PCAWG lung cancer RNA-sequencing data ranked by normalized enrichment score (NES), representing the enrichment of each cancer hallmark in PAH carriers compared to non-carriers. Hallmark signatures are ranked by *P*-values (at nominal *P* < 0.05). **c** Box plot showing the transcriptional score (TS) using an immune checkpoint-related gene set (*N* = 38) across three groups: *PAH* carrier in Lung-SCC PCAWG, *PAH* non-carrier in Lung-SCC PCAWG/TCGA, and normal lung samples from GTEx. A high TS indicates increased immune microenvironment correlation within a group

For a deeper functional analysis of OMIM genes as potential novel CPGs, we focused on phenylalanine hydroxylase (*PAH*; assigned to Cluster 2, ‘Tissue specific strong pLOF tolerant gene’) as it exhibited the highest frequency of pathogenic variants (PTVs and ClinVar pathogenic variants) among the 109 genes in the pan-cancer analysis (1.82%; 48 carriers out of 2,642 PCAWG cancer patients; Additional file 1: Table S2). *PAH* plays a pivotal metabolic role, converting L-phenylalanine to L-tyrosine. Inherited *PAH* variants result in conversion to L-phenylpyruvate (Fig. 5a) causing phenylketonuria (PKU), an autosomal recessive genetic disease with symptoms that include intellectual disability, seizures, nausea, and vomiting [55]. To gain a better understand of how *PAH* variants contribute to metabolic processes, we performed a metabolic simulation using a patient-specific genome-scale metabolic model (GEM) [39] (Additional file 2: Fig. S10a). Since *PAH* shows liver-specific expression in GTEx, PCAWG Liver-HCC samples (*N* = 312; including 3 *PAH* carriers and 309 *PAH* variant non-carriers) were applied to the model. The input data, defined as metabolite uptake rates, were obtained from GEM simulations (see Methods). As expected, the GEMs predicted that L-phenylalanine was more actively secreted (*P* = 1.42 × 10^−3^ by Student’s t-test) and actively converted to L-tyrosine in the *PAH* carriers than the non-carriers (Additional file 2: Fig. S10a).

To evaluate metabolic changes associated with pathogenic *PAH* variants, we conducted a CE-TOFMS-based metabolome analysis on 8 individuals heterozygous for the pathogenic *PAH* variants (from one parent of a PKU patient), and 22 healthy samples (i.e., *PAH* variant non-carriers without disease phenotype; see Methods and Fig. 5a). We annotated 220 peaks in the CE-TOFMS data using metabolite libraries. Intriguingly, PCA analysis using the 220 measured metabolite peaks clearly distinguished *PAH* variant carriers from non-carriers, indicating that *PAH* variants contribute to metabolic changes (Fig. 5a). There are 40 metabolites that showed significantly different concentrations in *PAH* variant carriers compared to healthy donors, including 8 up-regulated and 32 down-regulated metabolites (*P* < 0.05 by Welch’s T-test, Additional file 1: Table S9). As expected, we observed increased L-phenylalanine levels (Phe; 1.3-fold, *P* = 6.39 × 10^−4^ by Welch’s T-test) in the blood of *PAH* carriers compared to the non-carriers, which is a well-known characteristic of *PAH* deficiency.

Next, to understand the detailed tumorigenic mechanism of *PAH*, we selected Lung-SCC, which shows the highest frequencies of pathogenic variants in both PCAWG (4.3%) and TCGA (3.5%; Additional file 2: Fig. S10b). We also confirmed the prevalence of *PAH* pathogenic variants in Lung cancer using independent whole-exome sequencing (WES) data of Korean Lung-SCC samples (2.9%; 7 out of 245, Additional file 2: Fig. S10b). To test whether tumorigenesis depends on disease-linked *PAH* variants, we performed gene set enrichment analysis (GSEA) on the *PAH* carrier and non-carrier group using transcript expression levels from RNA-seq data of Lung-SCC PCAWG-TCGA (see Methods). Interestingly, immune-response related cancer hallmarks were strongly enriched in *PAH* carriers compared to non-carriers, including interleukin-2 and STAT5 signaling, inflammatory response, coagulation, and TNF-α signaling via NF-*k* (*P* = 9.00 × 10^−3^, *P* = 3.40 × 10^−2^, *P* = 2.10 × 10^−2^, and *P* = 4.90 × 10^−2^, respectively; Fig. 5b). Furthermore, we conducted a disease-based enrichment analysis by using 110 pre-defined metabolites (see Methods). The *PAH* dysfunction group shows the most significant association with inflammatory disease and myocardial infarction (*P* = 1.55 × 10^−8^, Additional file 2: Fig. S10c), followed by a metabolic disorder that affects weakened heart, muscle, and growth delay (3-Methylglutaconic aciduria type 2; *P* = 5.26 × 10^−8^). This indicates that metabolic changes linked to *PAH* pathogenic variants, in addition to causing PKU, could lead to multiple diseases, especially those associated with immune system dysfunction.

In addition, we evaluated the transcriptional score (TS), the summation of gene expression correlation coefficients between 38 immune checkpoint modulatory genes, within each group to assess the contribution of *PAH* variants to gene expression in the immune system (see Methods) [44]. We compared TSs across three groups: (i) *PAH* carrier (PTVs or ClinVar pathogenic variants; *N* = 15), (ii) *PAH* non-carrier in PCAWG-TCGA Lung-SCC (*N* = 478), and (iii) normal lung samples from GTEx [34] (*N* = 578). *PAH* pathogenic variant carriers (median TS = 19.14) demonstrated significantly higher TS compared to non-carrier (median TS = 14.69, *P* = 1.05 × 10^−3^ by Wilcoxon rank sum test) and normal lung samples (median TS = 9.25, *P* = 6.14 × 10^−8^ by Wilcoxon rank sum test, Fig. 5c). This indicates that the expression levels of immune check-point modulatory genes in *PAH* variant carriers tend to be more positively correlated with each other than non-carriers and normal lung samples, suggesting a potentially higher response rates to immune checkpoint inhibitors. Overall, our data suggest that *PAH* could modulate immune-associated pathways to contribute to cancer and that this gene is a potential new CPG.

## Discussion

We noted an enrichment of rare germline variants in autosomal recessive OMIM genes through our case–control analysis. Genetically, this might be associated with the potential for diverse clinical phenotypes in heterozygous carriers of rare germline variants. The prevailing assumption for heterozygotes with pathogenic variants is that carriers generally experience a normal lifespan, given that the wild-type allele maintains gene function [56]. However, with extended life expectancy, unexpected phenotypes attributable to rare germline variants may become evident in heterozygotes. For instance, heterozygous carriers might exhibit an altered metabolite profile or a lower penetrance that does not provoke classical disease phenotypes, including cancers [57].

Nonetheless, long-term exposure to this altered metabolism may adversely affect cellular fitness and could potentially trigger cancer.

In the four decades since Alfred Knudson proposed the ‘two-hit’ hypothesis, several classical cancer predisposition genes (CPGs) conforming to this model have been identified. These CPGs have been validated in specific tissues or in pan-cancer analyses [4, 58, 59]. Our study not only reaffirms the utility of the ‘two-hit’ hypothesis in classifying CPGs but also identifies exceptions where pathogenic variants are not linked to additional somatic alterations, even in known CPGs. Notably, genes in Cluster 2 exhibited low bi-allelic inactivation in the pan-cancer dataset along with significant tissue-specific gene expression levels, when compared to other clusters (mean tissue-specificity value in Cluster 2 = 0.87, other clusters = 0.58, *P* < 4.59 × 10^−9^ by Student’s t-test; Fig. 4d). This result might suggest the existence of a non-classical mechanism wherein bi-allelic inactivation is not commonly observed in pan-cancer analyses.

In our study, we highlight *PAH* as a novel candidate CPG that frequently harbors pathogenic variants in the pan-cancer dataset, and specifically in single-cancer types such as Lung-SCC. Our transcriptomic analysis reveals that Lung-SCC patients with pathogenic PAH variants demonstrate associations with immune-related pathways. This could be influenced by prolonged dysregulated metabolic stress [60, 61]. Indeed, patient-specific genome-scale metabolic models (GEMs) derived from PAH carriers reveal metabolic profiles that markedly deviate from those of the normal population (PAH non-carriers). These findings provide insights into a possible mechanism of cancer progression tied to altered metabolic profiles in individuals with pathogenic PAH variants. Importantly, comorbidity between phenylketonuria and various cancer types has been previously observed [62, 63], which further supports our findings.

In our study, we anticipate that sequencing an even larger collection of tumors will further refine these identified clusters and enhance our understanding of the diverse mechanisms contributing to cancer. Despite utilizing a well-balanced, large-scale cancer and control dataset, and controlling for population differences, our study still faces several limitations. First, although we employed a uniform pipeline for variant annotations in both case and control sets and observed no clear distinctions between sequencing centers in our PCA (Additional file 2: Fig. S2c-e), other potential technical factors remain unaddressed. These include sequencing kits, and age of samples. Such factors could influence the number of detected PTVs due to technical or biological discrepancies between the case and control sets. We observed nominal differences in the number of PTVs for genes that are neither CPGs nor OMIM genes (two-sample Kolmogorov–Smirnov test, *P* = 0.02). However, no such differences were discerned in CPGs (two-sample Kolmogorov–Smirnov test, *P* = 1; Additional file 2: Fig. S12). Given the limitations in the sample annotations for both the case and control datasets, a thorough confirmation remains technically challenging. Richer sample annotations in the future could bolster the regression model and more accurately quantify differences between case and control groups. The second potential limitation of this study arises from the unbalanced population composition, given that the PCAWG dataset is primarily derived from European/American patients (72.1%). This means that the full spectrum of pathogenic variants in non-European cancer patients may not have been entirely explored due to the limited sample size. For instance, we observed differences in the location of pathogenic variants of *PAH* between PCAWG-TCGA (mainly European) and Korean Lung-SCC patients, indicating potential ethnic-dependent variations in tumorigenesis mechanisms (Additional file 2: Fig. S10d). The sequencing of a larger cohort of both tumor samples and healthy control individuals will allow for further refinement and validation of PAH variant enrichment in Korean lung cancer data. Moreover, additional data will help elucidate the diverse roles of OMIM genes that contribute to cancer via different mechanisms.

## Conclusion

We prove a substantial excess of pathogenic variants in Mendelian disease-associated genes in comprehensive data from cancer patients compared to controls. This indicates that CPG-like OMIM genes could potentially increase the lifetime risk of cancer. Notably, our disease class-based enrichment analysis demonstrated significant enrichment of pathogenic variants in CPG-like OMIM genes associated with immune, neurological, and metabolic disorders. Indeed, immuno-oncology and onco-metabolism have emerged as crucial areas in oncology research over the past decade [64–67]. However, only a handful of germline variants associated with immuno-oncology and cancer metabolism have been identified as current therapeutic targets [68, 69]. We believe our findings offer novel strategies for understanding these new cancer hallmarks, specifically cellular metabolism and immune dysfunction, through the inherited variants of CPG-like OMIM genes [64].

### Supplementary Information

**Additional file 1:** **Table S1. **Sample information of the 2,642 PCAWG and 2,504 1KG in this study.** Table S2. **List of genes with pathogenic variant frequencies in Pan-cancer compared to controls.** Table S3. **Targeting cancer types for 6 significantly enriched cancer predisposition genes. **Table S4. **List of genes with pathogenic variant frequencies in single-cancer types compared to controls. **Table S5. **Functional annotation from GO and KEGG to 103 significantly enriched OMIM genes. **Table S6. **LOH events in 109 significantly enriched genes across 2,642 PCAWG samples. **Table S7. **109 significantly enriched genes in cases with four features. **Table S8. **Two-hit preference and tissue expression across single-cancer type. **Table S9. **PAH dependent 220 putative metabolites quantified from the CE-TOFMS measurement.

Additional file 2: **Figure S1. **Distribution of detected variants for case-control analysis. **Figure S2. **Distribution of population composition and principal components analysis (PCA) using common germline variants in PCAWG and 1KG. **Figure S3. **Comparison of enrichment of pathogenic variants in cancer compared to control between two different presenting approaches in a pan-cancer. **Figure S4.** *P*-value distribution from case-control analyses across single-cancer types. **Figure S5. **Enrichment of pathogenic variants in 2,642 cases compared to 2,504 control samples without logarithmic transformation. **Figure S6. **Independent case-control validation of CPG-like OMIM genes for the European population. **Figure S7. **Pathogenic variants enriched in diseases across pan-cancer and single-cancer types using the linear regression model. **Figure S8. **Distribution of copy-number alteration types between samples with LOH event and samples without LOH event. **Figure S9. **Gene clustering analysis. **Figure S10**. Possible carcinogenic mechanism mediated by PAH. **Figure S11. **Enrichment of rare (MAF 0.1%) pathogenic variants in cases compared to control samples. **Figure S12. **Distribution of the number of protein-truncating variants (PTVs).

## Data Availability

This paper re-analyses PCAWG whole genome sequencing (retrieved from http://dcc.icgc.org/pcawg/) [12], TCGA whole exome sequencing (https://cghub.ucsc.edu/) and 1000 genomes (http://www.internationalgenome.org/). All data sets are available upon request from the ICGC Data Access Compliance Office (DACO; http://icgc.org/daco), TCGA Data Access Committee (DAC) via dbGaP (https://www.ncbi.nlm.nih.gov/projects/gap/cgi-bin/study.cgi?study_id=phs000178.v11.p8) and the study authorization for 1,000 genomes (ftp://ftp.1000genomes.ebi.ac.uk/vol1/ftp/release/20130502/). The data set of rare variants is from the gnomAD Browser version 2.1.1 (https://gnomad.broadinstitute.org/) [20]. Gene expression patterns across multiple tissues, TAU score is downloaded from Palmer et al. (https://doi.org/10.18632/aging.202648) [33]. The scale of predicted loss-of-function constraint (LOEUF) is downloaded from Karczewski et al. (https://doi.org/10.1038/s41586-020-03174-8) [20]. Source code used for regression models are provided in a Github repository (https://github.com/SolipParkLab/OMIM_CPG) [70]. The metabolomics data have been deposited in the Zenodo (https://doi.org/10.5281/zenodo.6791873) [71]. The sequencing data supporting the conclusions of this article (Korean Lung cancer) are deposited in the NCBI Sequence Read Archive (SRA) repository are available (https://www.ncbi.nlm.nih.gov/sra/?term=PRJNA855112).

## Abbreviations

CPG: Cancer predisposition gene
OMIM: Online Mendelian Inheritance in Man
PCAWG: Pan-cancer Analysis of Whole Genomes project
1KG: 1000 Genome project
TCGA: The Cancer Genome Atlas
ICGC: International Cancer Genome Consortium
VCF: Variant Call Format
MAF: Minor Allele Frequency
AFR: African
SAS: South Asian
EAS: East Asian
AMR: Latino/Admixed American
NFE: Non-Finnish European
FIN: Finnish European
ASJ: Ashkenazi Jewish
gnomAD: The Genome Aggregation Database
PCA: Principal component analysis
PTV: Premature protein truncation variants
AR: Autosomal-recessive gene
AD: Autosomal-dominant gene
GLM: Generalized linear regression model
DAVID: Database for Annotation, Visualization and Integrated Discovery
CNA: Copy-number alteration
LOH: Loss-of-heterozygosity
LOEUF: Loss of function observed/expected upper bound fraction
pLoF: Predicted for loss-of-function
TS: Transcriptional score
WES: Whole exome sequencing
SOD: Somatic driver gene
SNV: Single nucleotide variant
FDR: False discovery rate
GEM: Genome-scale metabolic model
