# Supplementary material for: Systematic analysis of Mendelian disease-associated gene variants reveals new classes of cancer-predisposing genes
Source: Genome Med. 2023 Dec 25;15:107. doi: 10.1186/s13073-023-01252-w (PMC10749499; doi:10.1186/s13073-023-01252-w)
Supplement: Supplementary file 2 — Additional file 2: Figure S1. Distribution of detected variants for case-control analysis. Figure S2. Distribution of population composition and principal components analysis (PCA) using common germline variants in PCAWG and 1KG. Figure S3. Comparison of enrichment of pathogenic variants in cancer compared to control between two different presenting approaches in a pan-cancer. Figure S4. P-value distribution from case-control analyses across single-cancer types. Figure S5. Enrichment of pathogenic variants in 2,642 cases compared to 2,504 control samples without logarithmic transformation. Figure S6. Independent case-control validation of CPG-like OMIM genes for the European population. Figure S7. Pathogenic variants enriched in diseases across pan-cancer and single-cancer types using the linear regression model. Figure S8. Distribution of copy-number alteration types between samples with LOH event and samples without LOH event. Figure S9. Gene clustering analysis. Figure S10. Possible carcinogenic mechanism mediated by PAH. Figure S11. Enrichment of rare (MAF 0.1%) pathogenic variants in cases compared to control samples. Figure S12. Distribution of the number of protein-truncating variants (PTVs). [file 13073_2023_1252_MOESM2_ESM.docx]

**Additional file 2: Supplementary Figures**

**
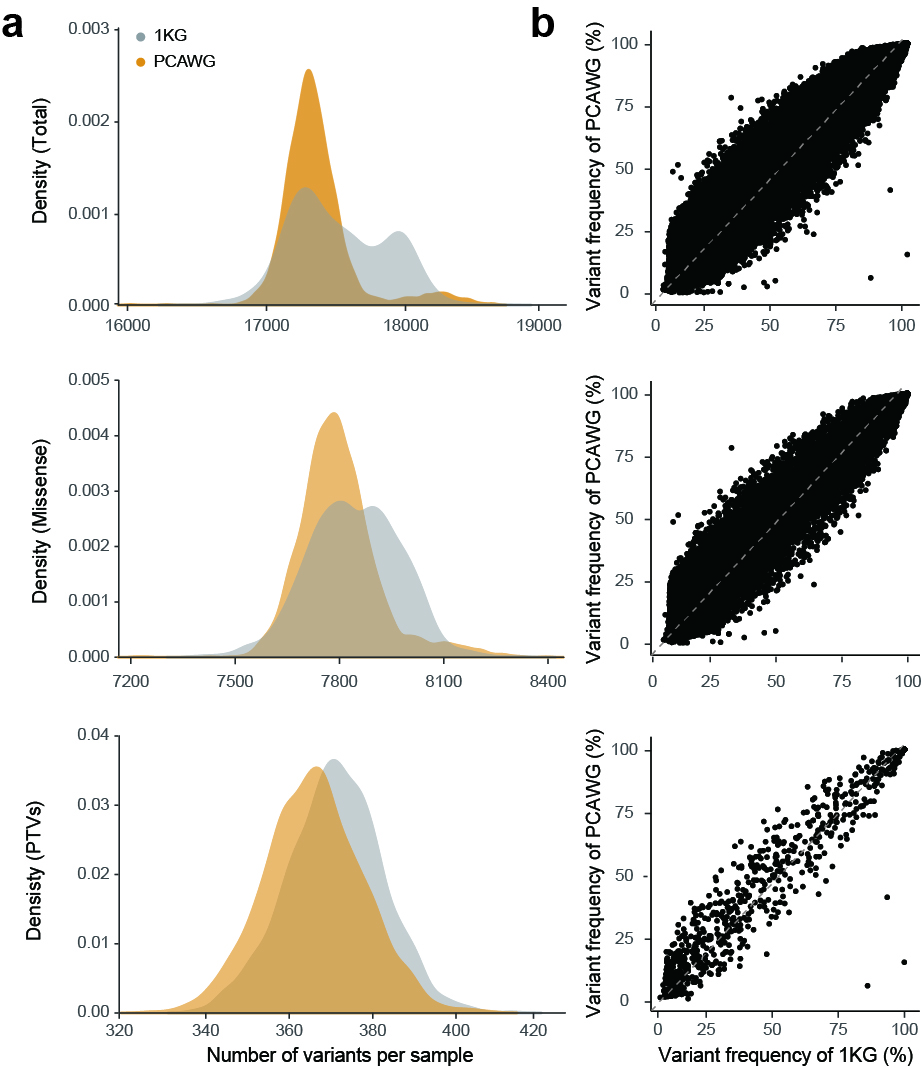
**

**Fig S1. Distribution of detected variants for case-control analysis.** (**a**) The number of detected variants across three types per sample was compared between PCWAG and 1000 Genomes Project (1KG). Density plots were colored to represent each cohort (grey: 1KG, yellow: PCAWG). (**b**) The correlation of variant frequencies between PCAWG and 1KG for total variants, missense variants, and protein-truncating variants (PTVs). The grey dashed line indicates a slope = 1.

**
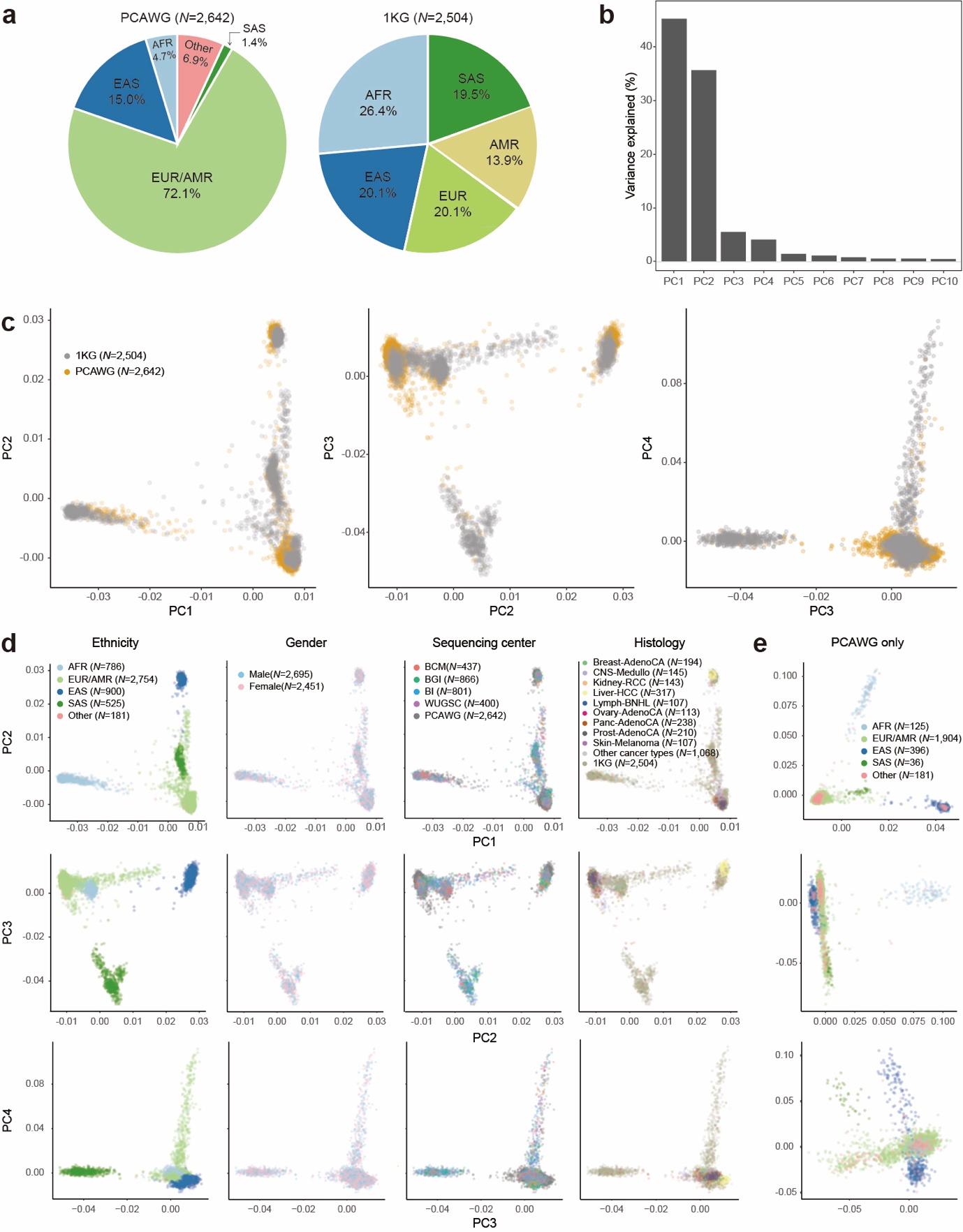
**

**Fig S2. Distribution of population composition and principal components analysis (PCA) using common germline variants in PCAWG and 1KG.** (**a**) Population composition in PCAWG and 1KG. AFR: African, AMR: American, EAS: East Asian, EUR: European, and SAS: South Asian. (**b**) The percentage of variance explained by each PCA component. The first four components account for approximately 90% of the total variance. (**c**) PCA was performed using common nonsynonymous variants (population allele frequency > 5% in gnomAD exome v2) in PCAWG and 1KG samples. Samples were colored based on the data source. (**d**) PCA plots using PCAWG and 1KG samples, with colors representing self-reported ethnicities, clinical information (gender, histology) and technical covariate of samples (sequencing center). (**e**) PCA plots using only PCAWG samples for two-hit preference analysis. Samples were colored based on self-reported ethnicities.

**
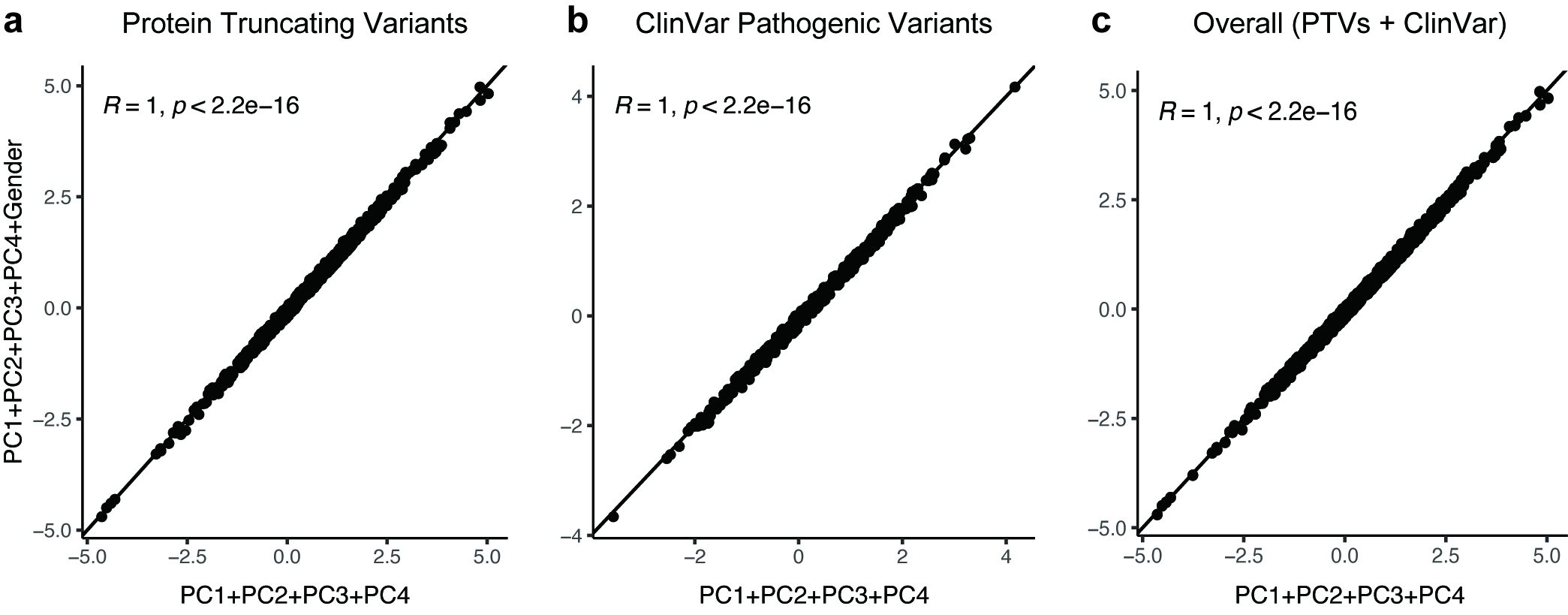
**

**Fig S3.** Comparison of enrichment of pathogenic variants in cancer compared to control, presenting log2(odds ratio), between two different regression models in a pan-cancer: The x-axis represents the model after controlling for population differences in case-control samples, while the y-axis represents the model that controls both for population differences and gender in case-control samples. This is depicted across three distinct pathogenic classes: (**a**) PTVs, (**b**) ClinVar pathogenic variants, and (**c**) a combination of PTVs and ClinVar pathogenic variants.


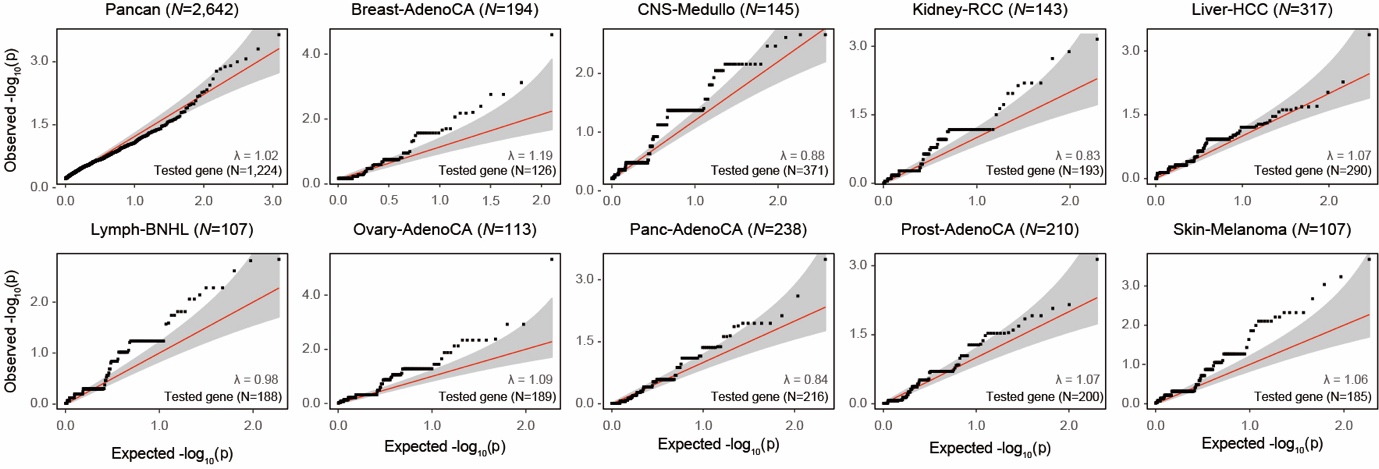


**Fig S4. *P*-value distribution from case-control analyses across single-cancer types.** Quantile-quantile (Q-Q) plots of *P*-values from the case-control analysis, showing the enrichment of pathogenic variants in case samples compared to control samples. The shaded area indicates the 95% confidence interval. The sample size for each cancer type is presented on the top, the number of tested genes is shown, and the values of the inflation factor λ are displayed on the bottom.

**
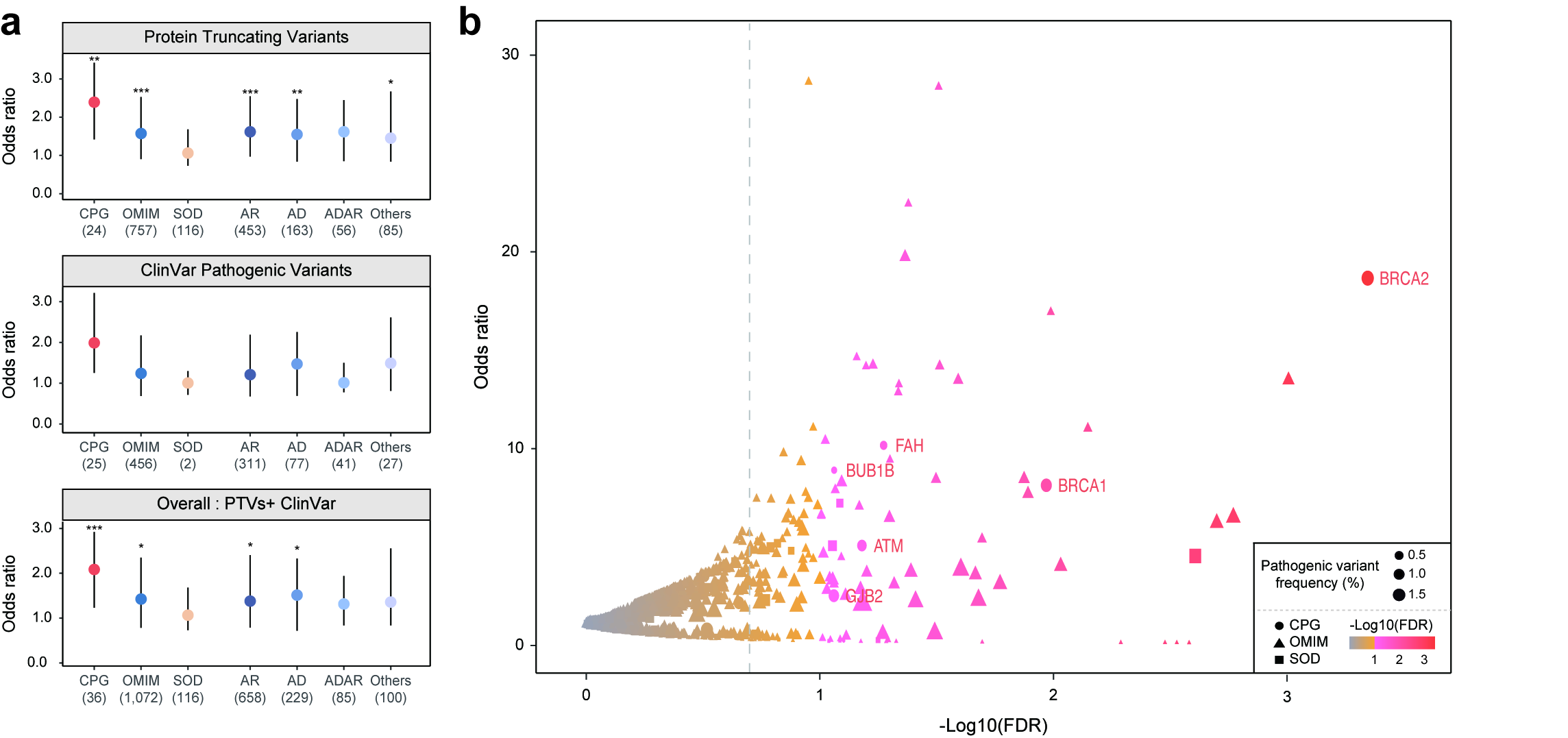
**

**Fig S5. Enrichment of pathogenic variants in 2,642 cases compared to 2,504 control samples without logarithmic transformation.** **(a)** Enrichment of pathogenic variants in cases compared to control samples for three gene sets and four OMIM subgroups (* *P* < 0.05, ** *P* < 0.01, *** *P* < 0.001). The median value of each gene set is displayed as a circle. The length of each whisker represents 1.5 times the interquartile range (shown as the height of each box). **(b)** Excess of pathogenic variants (PTVs and ClinVar pathogenic variants) in case samples compared to control samples for 1,265 individual genes. The dashed vertical line represents the statistical significance threshold (*FDR*=20%). Color indicates significance, shape represents the type of gene, and size presents the frequency of pathogenic variants.

**
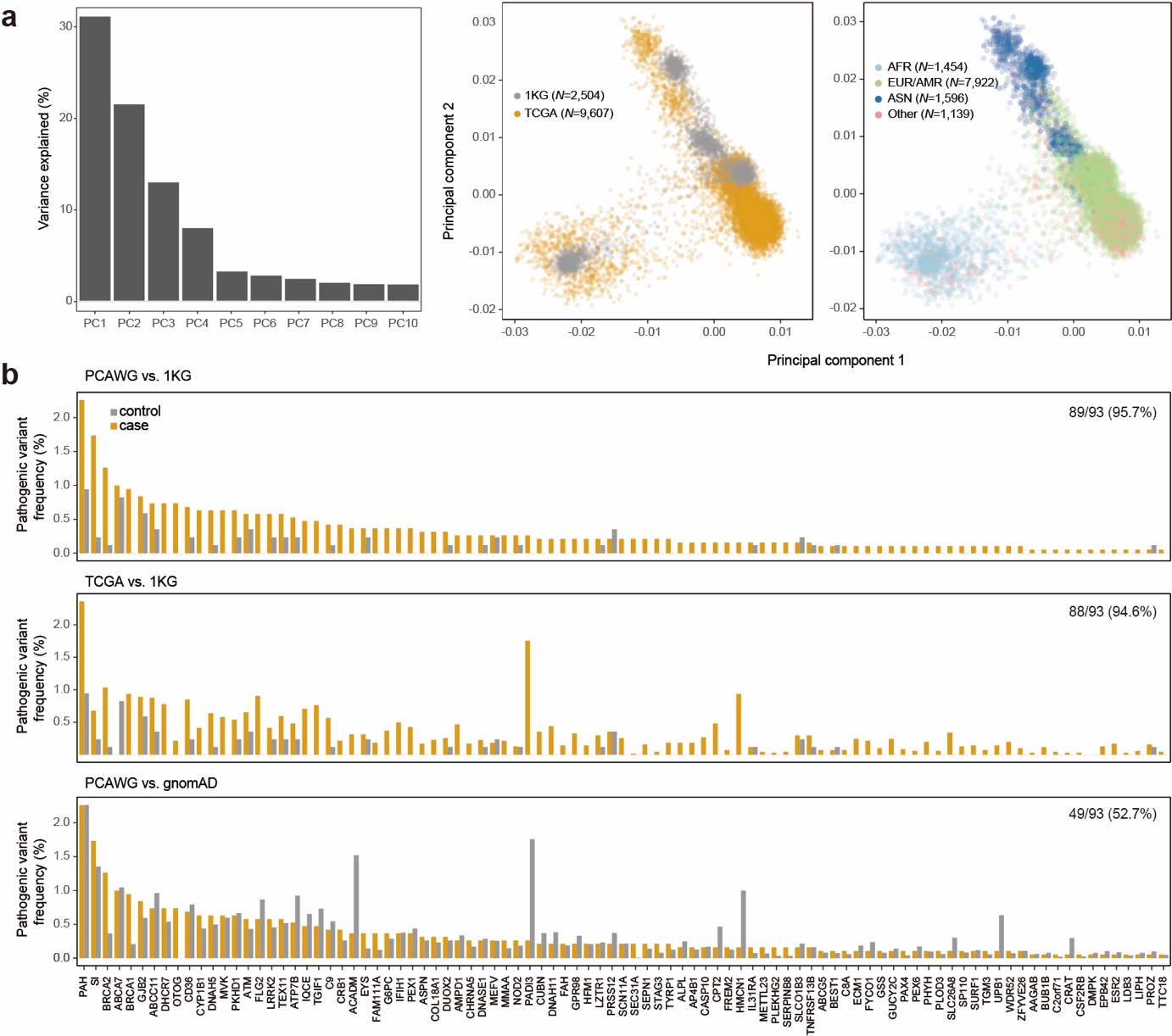
**

**Fig S6. Independent case-control validation of CPG-like OMIM genes for the European population.** (**a**) Principal component analysis (PCA) was performed using common variants (population allele frequency > 5% in gnomAD exome v2) in TCGA and 1KG samples. The percentage of variance explained by each PCA component is shown for TCGA and 1KG samples. Samples were colored based on the data sources and self-reported ethnicities. (**b**) The frequency of pathogenic variants (rare PTVs or ClinVar variants; y-axis) in 93 CPG-like OMIM genes detected in case (PCAWG)-control (1KG) analysis at an FDR 20% is shown (93 out of 109 CPG-like OMIM genes were validated in European population-based analysis). The top panel compares the frequency of pathogenic variants in cancers with PCAWG_European samples to 1KG_European samples. The middle panel compares the frequency of pathogenic variants in cancers with TCGA_European samples to 1KG_European samples. The bottom panel compares the frequency of pathogenic variants in cancers with PCAWG_European samples to gnomAD_European Noncancer ancestry.


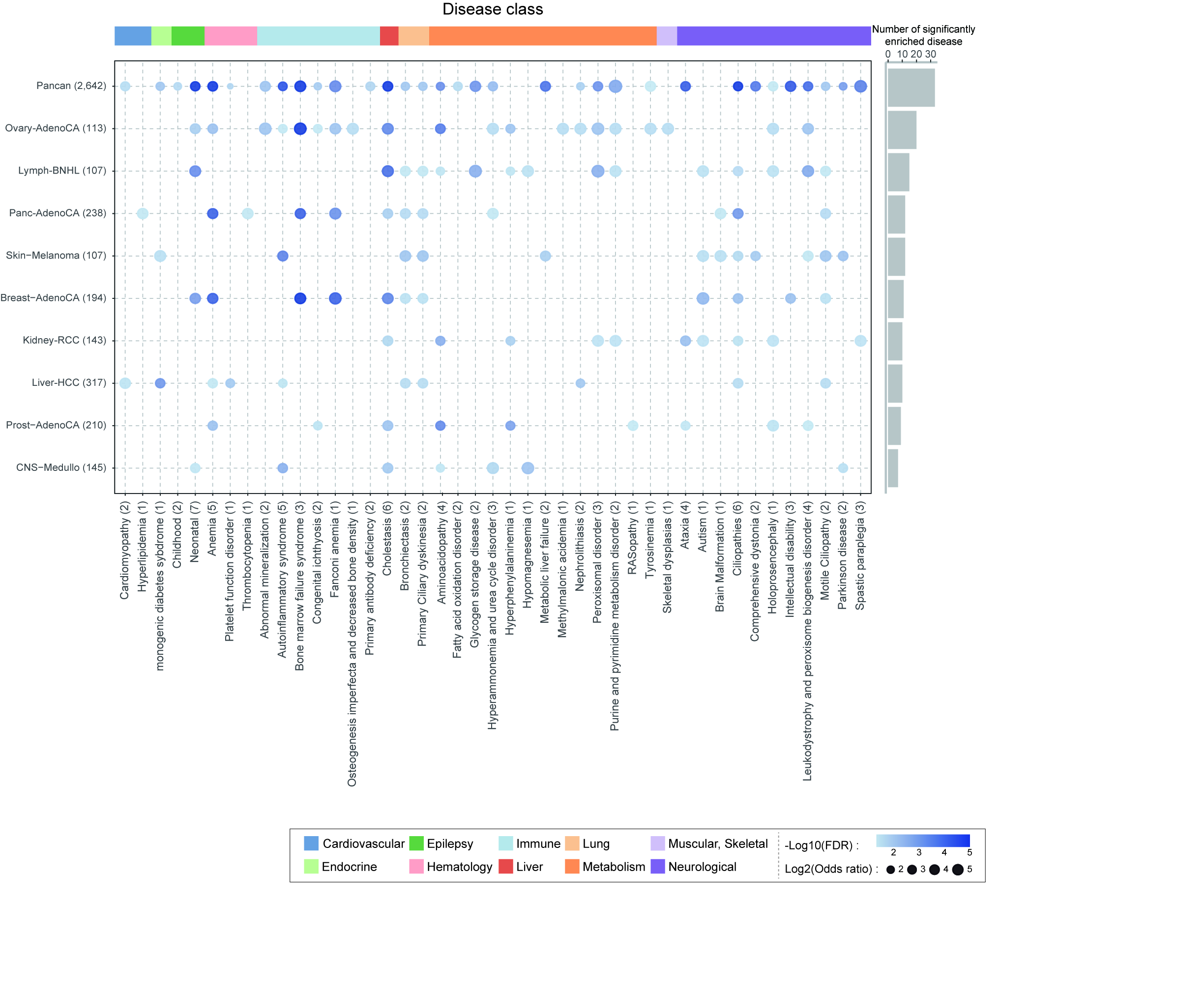


**Fig S7. Pathogenic variants enriched in diseases across pan-cancer and single-cancer types using the linear regression model.** Enrichment of pathogenic variants was analyzed using 1,487 disease-associated genes, which includes 463 newly identified genes from the Clinical Genome Resource. Circle size indicates the excess of pathogenic variants per gene set, and color represents the significance of the enrichment in a specific cancer type compared to the control (1KG). Numbers in parentheses indicate the number of samples in each cancer type (left) and the number of tested genes in each disease (bottom). The bar plot on the right shows the number of detected diseases in each cancer type at a false discovery rate (FDR) of less than 0.05.


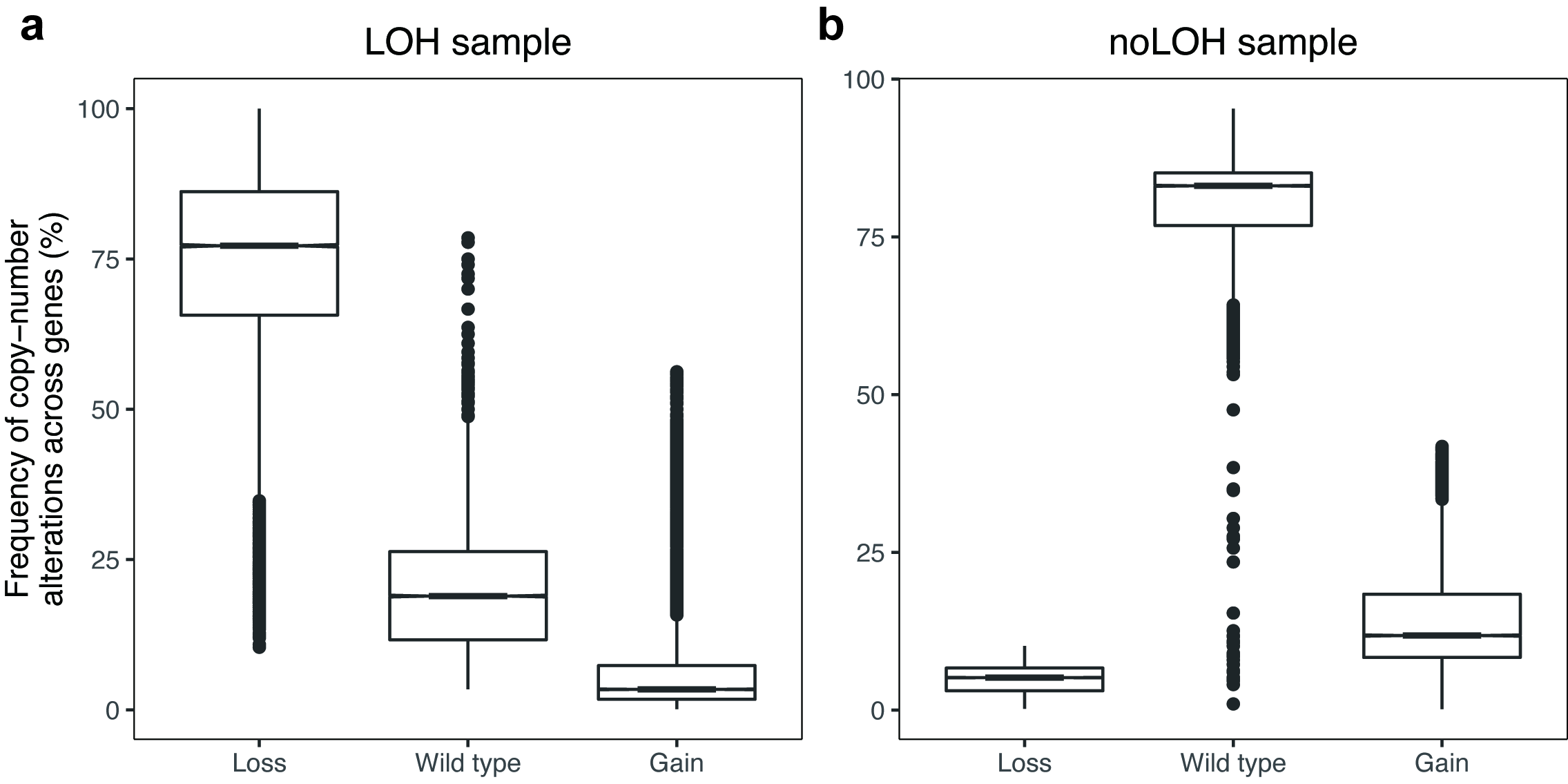


**Fig S8. Distribution of copy-number alteration types between (a) samples with LOH event and (b) samples without LOH event.**

**
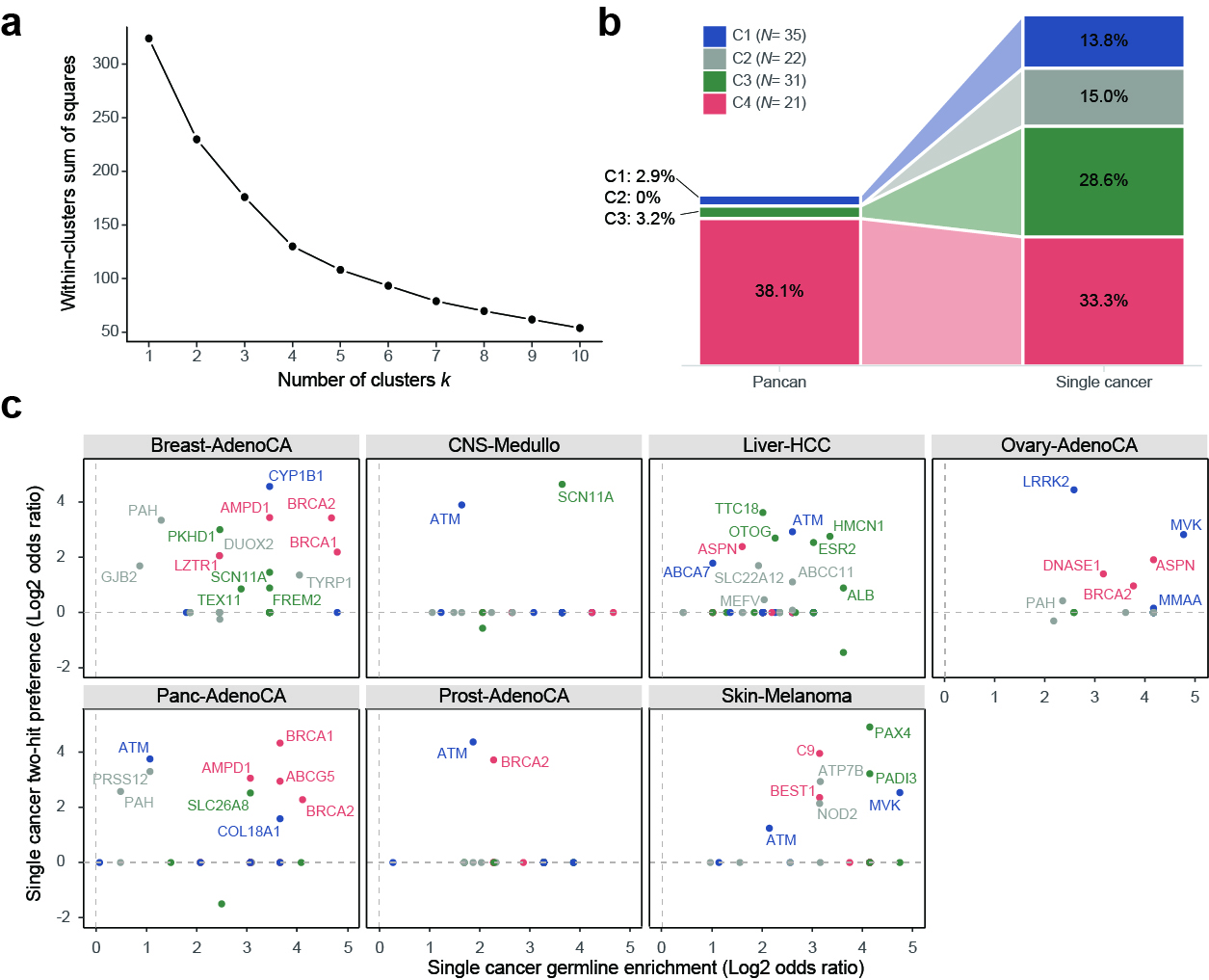
**

**Fig S9. Gene clustering analysis.** (**a**) A scree plot is shown to determine the optimal number of clusters using the enhanced *k*-means clustering algorithm (*k*=4). (**b**) The frequency of two-hit preferable genes at an FDR 20% is shown across clusters in the Pan-cancer analysis and single cancer types. (**c**) The odds ratio (Log2) from a case-control analysis across single cancer types is compared to two-hit preferences (Log2), representing the excess of pathogenic variants in samples with loss of heterozygosity (LOH) compared to samples without LOH. Genes were colored based on their assigned clusters as shown in (**b**).


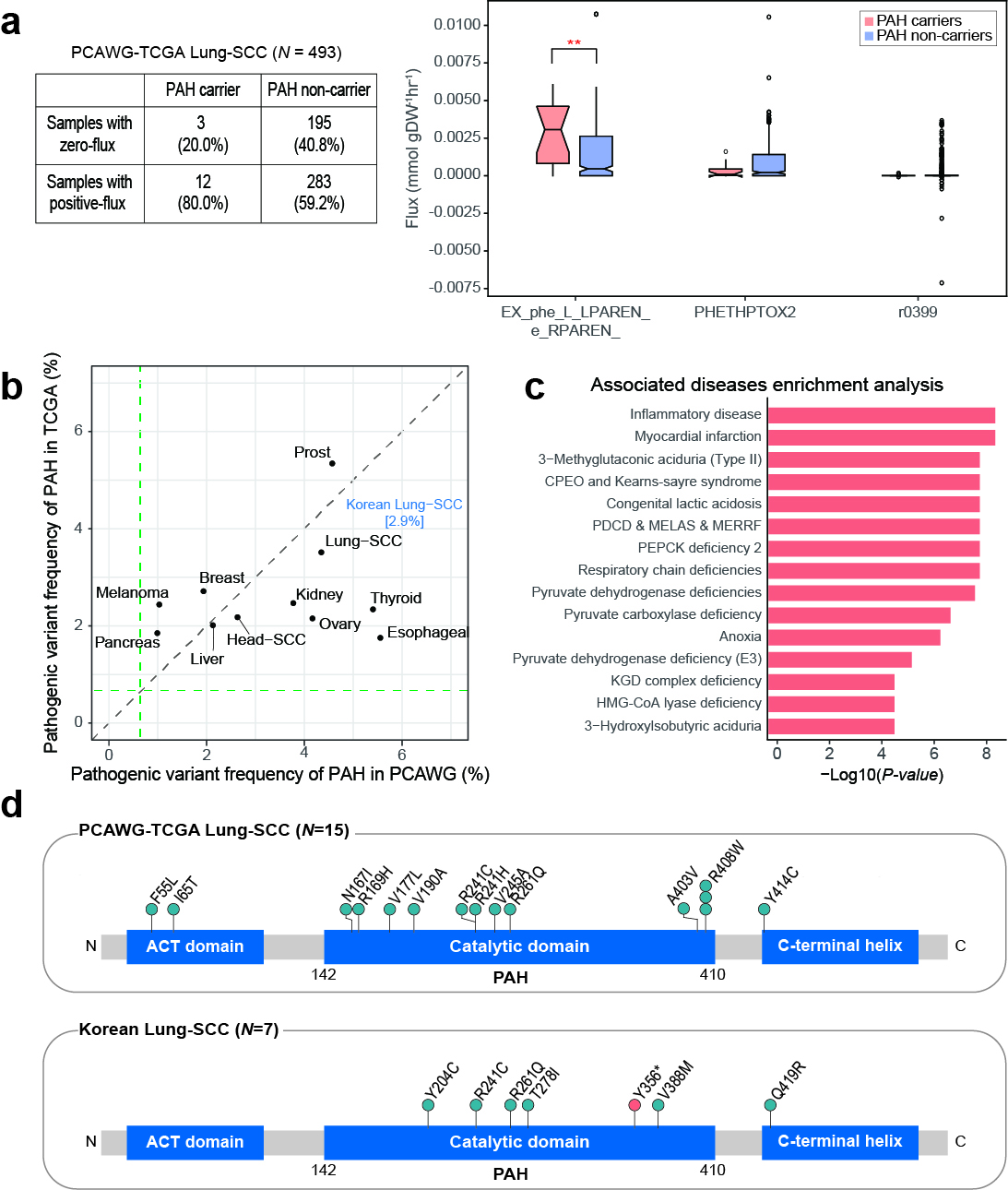


**Fig S10. Possible carcinogenic mechanism mediated by PAH.** (**a**) The left panel shows the results of metabolic simulation for PAH carriers and non-carriers using genome-scale metabolic models (GEMs) of the corresponding PCAWG-TCGA Lung-SCC samples, summarized in the table. The right panel presents a box plot of the predicted flux of the L-phenylalanine exchange reaction ('EX_phe_L_LPAREN_e_RPAREN_' in Recon 2M.2) and two additional reactions ('PHETHPTOX2' and 'r0399') related to PAH. The asterisks indicate significant differences (* *P* < 0.01). The greater the value of the L-phenylalanine exchange reaction means the more active secretion of L-phenylalanine. In addition, the greater values of 'PHETHPTOX2' and 'r0399' in PAH non-carriers indicate that L-phenylalanine is more actively converted to L-tyrosine than PAH carriers. (**b**) Metabolic disease enrichment in PAH carriers (*N*=8) compared to non-carriers (*N*=22) of Korean samples is shown. Significantly enriched metabolic diseases are presented (*P* < 0.05 by Fisher’s exact test). CPEO: chronic progressive external ophthalmoplegia, PCDC: pyruvate dehydrogenase complex deficiency, MELAS: mitochondrial encephalopathy, lactic acidosis, and stroke-like episodes, MERRF: myoclonic epilepsy with ragged-red fibers, PEPCK: Phosphoenolpyruvate carboxykinase, KGD: 2-ketoglutarate dehydrogenase, and HMG-CoA: 3-Hydroxy-3-methylglutaryl-CoA lyase. (**c**) The frequencies of *PAH* pathogenic variant across cancer types in PCAWG (x-axis) and TCGA (y-axis; after removing overlapping samples with PCAWG) are shown. The pathogenic variant frequency of *PAH* in an independent cohort, Korea Lung cancer carcinoma (2.9%), is presented in blue. The green dashed lines represent the *PAH* frequency in 1KG (0.7%). (**d**) Lollipop plots show the pathogenic variants in *PAH* using the Lung-SCC (PCAWG-TCGA) in the top panel and the Korean Lung-SCC in the bottom panel. Blue circles indicate missense variants and red circle represents protein truncating variants. The number of the circle indicates the number of detected variants in each cohort (Y-axis).


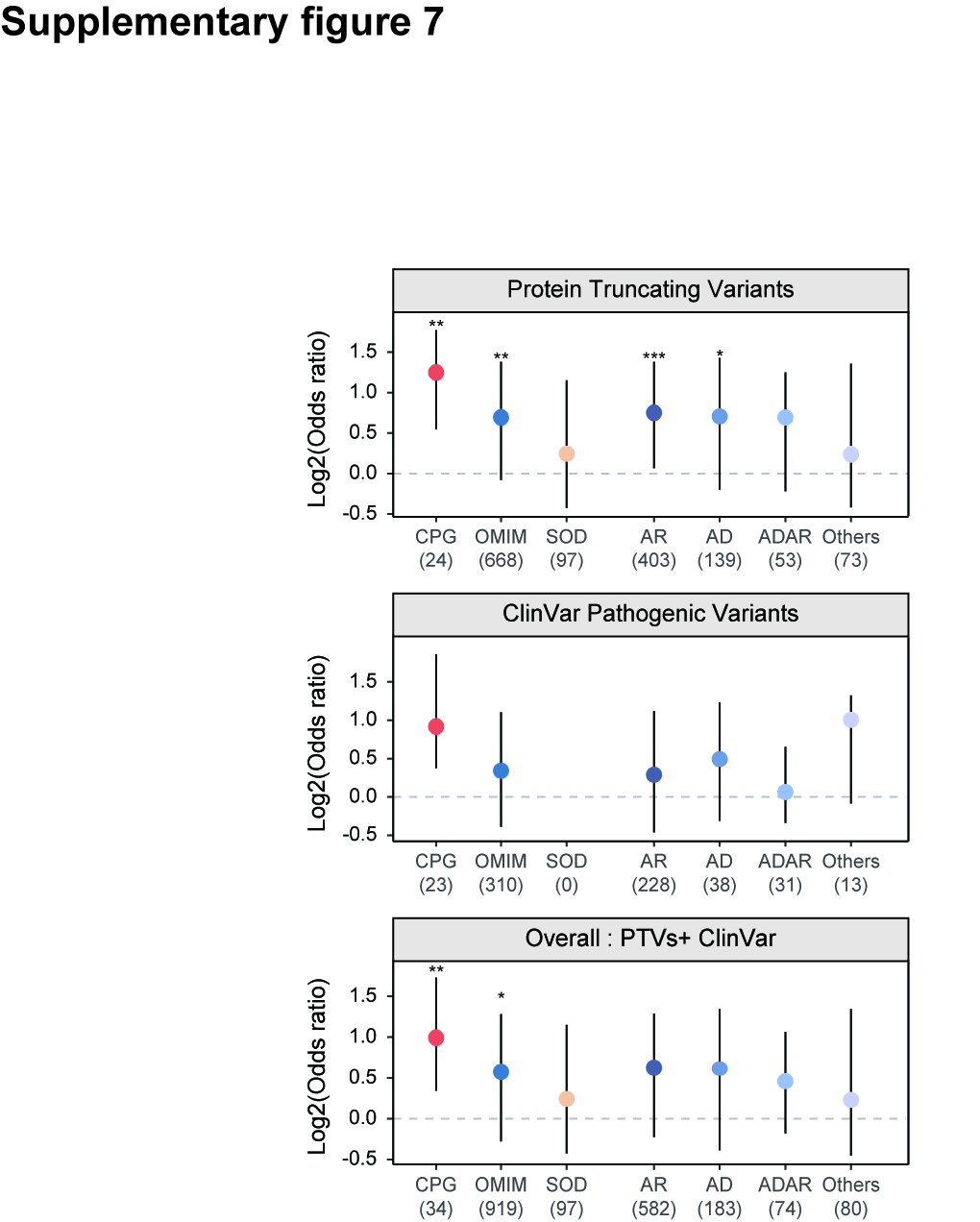


**Fig S11. Enrichment of rare (MAF 0.1%) pathogenic variants in cases compared to control samples.** The median value of each gene set is displayed as a circle. The length of each whisker is 1.5 times the interquartile range, shown as the height of each box.

**
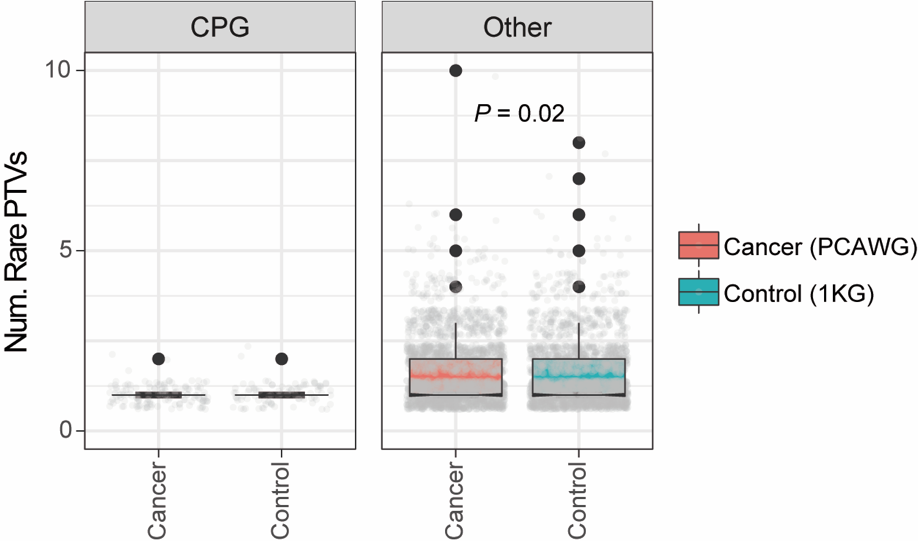
**

**Fig S12. Distribution of the number of protein-truncating variants (PTVs) in CPGs and other genes (that are neither CPGs nor OMIM-associated).** The median value of each gene set is displayed as a circle. The length of each whisker is 1.5 times the interquartile range, shown as the height of each box.
